# Antecedents of unfinished nursing care: a systematic review of the literature

**DOI:** 10.1186/s12912-022-00890-6

**Published:** 2022-06-14

**Authors:** Stefania Chiappinotto, Evridiki Papastavrou, Georgios Efstathiou, Panayiota Andreou, Renate Stemmer, Christina Ströhm, Maria Schubert, Susanne de Wolf-Linder, Jessica Longhini, Alvisa Palese

**Affiliations:** 1 University of Tor Vergata, Roma, Italy; 2 Department of Nursing, School of Health Sciences, Cyprus University of Technology, Limassol, Cyprus; 3 Nursing Services, Ministry of Health, Nicosia, Cyprus; 4 Medical School, University of Nicosia, Nicosia, Cyprus; 5 Catholic University of Applied Sciences, Mainz, Germany; 6 School of Health Professions, Zurich University of Applied Science, Winterthur, Switzerland; 7 Department of Medical Sciences, University of Udine, Udine, Italy

**Keywords:** Antecedents, Factors, Implicit of nursing care, Missed nursing care, Predictors, Reasons, Systematic review, Tasks left undone, Unfinished nursing care

## Abstract

**Background:**

Unfinished Nursing Care (UNC) concept, that express the condition when nurses are forced to delay or omit required nursing care, has been largely investigated as tasks left undone, missed care, and implicit rationing of nursing care. However, no summary of the available evidence regarding UNC antecedents has been published. The aim of this study is to identify and summarise antecedents of UNC as documented in primary studies to date.

**Methods:**

A systematic review according to the Preferred Reporting Items for Systematic Reviews and Meta-Analyses guidelines was conducted. MEDLINE, CINAHL, SCOPUS, and PROSPERO databases were searched for quantitative studies reporting the relationships between antecedents and UNC published after 2004 up to 21 January 2020. The reference lists of secondary studies have been scrutinised to identify additional studies. Two reviewers independently identified studies and evaluated them for their eligibility and disagreements were resolved by the research team. The quality appraisal was based on the Joanna Briggs Institute Critical Appraisal tools, according to the study designs. A data extraction grid was piloted and then used to extract data. The antecedents that emerged were thematically categorised with an inductive approach.

**Results:**

Fifty-eight studies were included; among them, 54 were cross-sectional, three were cohort studies, and one was a quasi-experimental study. They were conducted mainly in the United States and in hospital settings. The UNC antecedents have been investigated to date at the (a) unit (e.g., workloads, non-nursing tasks), (b) nurse (e.g., age, gender), and (c) patient levels (clinical instability).

**Conclusions:**

At the unit level, it is highly recommended to provide an adequate staff level, strategies to deal with unpredictable workloads, and to promote good practice environments to reduce or minimise UNC. By contrast, at the nurse and patient levels, there were no clear trends regarding modifiable factors that could decrease the occurrence of UNC. The map of antecedents that emerged can be used to design interventional studies aimed at changing research from merely descriptive to that which evaluates the effectiveness of interventions.

**Supplementary Information:**

The online version contains supplementary material available at 10.1186/s12912-022-00890-6.

## Background

Unfinished Nursing Care (UNC) is an overarching term encompassing several concepts [1] that express the condition when nurses are forced to delay or omit required nursing care [2]. The various concepts included in UNC have been largely conceptualised both theoretically [3, 4] and empirically by validating instruments measuring the occurrence of the phenomenon, namely the Tasks Left Undone Scale [5], the Basel Extent of Rationing of Nursing Care instrument [3], the MISSCARE Survey [6], and the Perceived Implicit Rationing of Nursing Care survey instrument [7]. Moreover, several studies have established outcomes associated with UNC both at the patient (e.g., falls, hospital-acquired infections, pressure ulcers) and at the nurse level (e.g., job satisfaction, intention to leave) [8, 9]. However, to inform decisions regarding which interventions should be implemented to minimise and/or reduce UNC [10], more studies about UNC antecedents have been recommended [11]. Above all, sound systematizations of the available evidence base on factors contributing to providing high quality nursing care or posing barriers in providing the care needed for shaping and optimizing nursing care are need. Despite the impetus reported in this research area [12], no summary of the available evidence about UNC antecedents has been produced to date: therefore, the primary intent of this study was to fill this gap.

### Antecedents of unfinished nursing care

Within the overarching UNC term [1], there are three main concepts: Tasks Left Undone, Missed Nursing Care, and Implicit Rationing of Nursing Care. Tasks Left Undone was first conceptualised by Solchalski in 2004 [4], defined it as activities left unfinished during the last shift because nurses lacked the time to undertake them. In this context, nurse workloads and time constrains were both considered antecedents; however, specific factors triggering or hindering tasks left undone were not conceptualized [4].

A few years later, Kalisch [13] introduced the concept of Missed Nursing Care as every aspect of nursing care required by a patient that is partly or totally omitted or significantly delayed. In the first theoretical model, four elements at the nurse level were related to Missed Nursing Care: team norms, decision-making processes, internal values and beliefs, and habits [14]. In the same year, Kalisch and Williams [6] developed the MISSCARE survey to measure Missed Nursing Care. This instrument also includes a set of other reasons of missed care as perceived by nurses, namely deficiencies in communication, material resources, and labour resources. A few years later, Kalisch and Xie [15] advanced their model by including three additional antecedents regarding (a) the hospital (size, teaching status, Magnet status), (b) the unit(s) (case mix index, nurse staffing levels, type of nurse staffing, absenteeism, overtime, and work schedules), and (c) the teamwork characteristics. In the same period, Schubert et al. [3] established the Implicit Rationing of Care concept as the withholding of or failure to carry out necessary nursing measures for patients. In their theoretical model, antecedents considered (a) the organizational variables (namely the budget, policy priorities, resource allocation, the management structure, the culture, and the climate); (b) the nursing work environment variables, including the adequacy of resources and skill mix, interdisciplinary collaboration, nursing management, autonomy, and responsibility; and (c) the philosophy of care variables, such as the priority setting, the cultural values, the standards of care, and local and national guidelines. Moreover, patient variables, including illness severity and co-morbidities, and nurse variables, such as the nurses’ experience(s), education, skills, and knowledge have been established as other factors affecting the occurrence of Implicit Rationing of Nursing Care [3].

According to the state of the research in this field, UNC antecedents were considered in a linear relationship [4, 14], within an input–process–outcome model [16]. In more recent years, the debate has moved to more complex models, the so-called systems approach [16], where UNC has been examined holistically rather than as the sum of different parts. In this context, researchers have considered several factors as interrelated each other [3]. For example, hospital units comprising different sub-systems interacting with each other, are influenced by the nursing philosophy and the work environment that might be different in each of them [3]. Alongside these internal interactions, external factors might affect each unit, as for example, the hospital’s organisational variables [3]. Therefore, a multi-level approach has been introduced in this research debate, examining how upper-level management might affect the clinical nurses and, consequently, the UNC occurrence at the bedside [16]. Thus, factors external to the unit at the hospital, regional, or national levels (e.g., policies, rules) as implementing cost-containing measures in the attempt to increase productivity and efficiency, might affect the UNC occurrence at the bedside [3, 16].

## Methods

The following research questions were addressed: (a) What antecedents have been investigated to date as associated with the UNC? (b) What is the direction of the relationships between such antecedents and the UNC that has been documented to date?

Therefore, the aims of the study were to (a) map factors, predictors, correlates, or linked factors – hereafter, ‘antecedents’, and (b) summarise the direction of their relationships with UNC. A systematic review of the literature was performed according to the Preferred Reporting Items for Systematic Reviews and Meta-Analyses guidelines [17].

### Sources

The Sample, Phenomenon of Interest, Design, Evaluation, Research type (SPIDER) [18] methodology was used to establish the review question. Then, according to the elements specified (Table 1), the search terms were identified [19] (Table 2) without considering specific key words as expressing the influence of specific factors (e.g., morning shifts) [20].

**Table 1:** SPIDER Specifications [18]

**Sample**	Registered Nurses
Phenomenon of interest	Unfinished Nursing Care, Missed Nursing Care, Rationed Nursing Care
Design	Quantitative, cross-sectional, longitudinal, retrospective, case–control, experimental, or quasi-experimental studies
Evaluation	All reported antecedents, predictors, risk factors, correlated factors
Research type	Quantitative

*Abbreviation: SPIDER* Sample, Phenomenon of Interest, Design, Evaluation, Research type

**Table 2:** Key terms used in the search strategy

Key terms for UNC	Implicit Rationed Nursing Care
	Implicit Rationing
	Missed Care
	Missed Nursing Care
	Omitted Nursing Care
	Rationed Care
	Task Left Undone
	Task(s) Undone
	Unfinished Care
Key terms for antecedents	Antecedents
	Causes
	Determinants
	Factors
	Predictors
	Reasons
	Related/correlated factors

*Abbreviation: UNC* Unfinished Nursing Care

In a preliminary phase, the International Prospective Register of Systematic Reviews (PROSPERO) database was checked to determine whether there were ongoing systematic reviews about the antecedents of UNC. Then, MEDLINE, the Cumulative Index to Nursing and Allied Health Literature (CINAHL), and SCOPUS were searched.

### Search strategy

There were included those primary studies: (a) investigating antecedents of the UNC in adult care settings (patients ≥ 18 years of age); (b) providing measures of relationships between the investigated antecedent(s) and the occurrence of the UNC; (c) employing quantitative designs and reporting the abstract, (d) published in English, German, Greek or Italian, the languages accessible to the research team (see authors); (e) from 2004 up to 21 January 2020, when the Task Left Undone concept, included in the UNC overarching term, was first established [4, 21]. Reviews, systematic reviews, overviews, or integrative reviews retrieved were scrutinised in their references manually for relevant primary studies potential eligible according to the inclusion criteria.

Specifically, there were included those studies providing inferential statistics such as correlations, associations (odds ratios, relative risks), or other estimations (e.g., beta) to evaluate the relationship between one or more antecedents and the occurrence of the UNC. In some studies, the authors did not provide sufficient data to establish how the investigated variables were associated with UNC [22]. In these cases, the available conceptual models of the UNC were used to assess the study’s eligibility [3, 14, 23].

There were excluded those studies: (a) qualitative in nature; (b) regarding settings caring for patients < 18 years (paediatric) and other settings (obstetrics and psychiatric) due to specific care provided and the relevance of other potential factors in hindering/increasing UNC (e.g. the role of family relatives) [21]; (c) including other health care professionals (e.g., midwives), (d) reporting only descriptive measures of antecedents (e.g., frequencies), thus not assessing associations with UNC; (e) not reporting an abstract and published in languages other than those previously listed. Reviews were also excluded, although their reference lists were checked manually for appropriate studies.

### Selection and data extraction

**Fig. 1:**
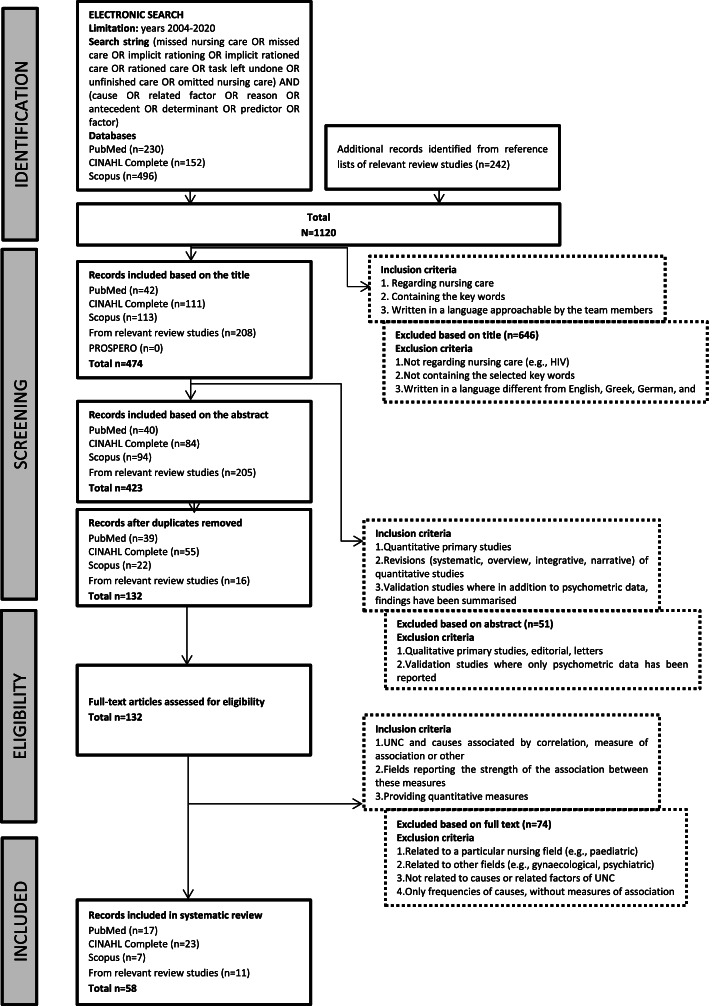
Flow diagram of included studies. Abbreviations: CINAHL, Cumulative Index to Nursing and Allied Health Literature; PRISMA, Preferred Reporting Items for Systematic Reviews and Meta-Analyses; PROSPERO, Prospective Register of Systematic Review; UNC, Unfinished Nursing Care

A total of 1,120 sources were identified (Fig. 1). Subsequently, 990 studies, including 291 duplicates, were excluded by two researchers’ experts in the unfinished care field, who evaluated titles and abstracts independently and then agreed upon which studies to evaluate further. The remaining 132 studies were carefully read in their full texts by the same researchers, in an independent fashion. Disagreements emerged regarding four studies; thus, the entire research team (see authors) was involved in multiple meetings, in order to reach consensus regarding the inclusion. At the end of the process, 58 studies were included.

Two researchers developed the data extraction grid by including the following elements: author(s); country; aims; design (if longitudinal: assessment points); when the study was performed (year(s); the setting (hospital, community, number of units or centres involved); the sampling methods used; the participants involved and their main demographic characteristics; the instruments used for data collection (explanatory variables [as antecedents] and the UNC phenomenon); and the main findings regarding the relationships between the antecedent(s) and the occurrence of UNC.

The entire research team then approved the extraction grid via an online meeting. Three researchers piloted the grid independently with three studies, and all authors agreed that no changes were needed at the end of the pilot. Then, the same three researchers independently extracted the data and agreed upon.

The data extracted were thematically analysed [24] using an inductive approach [25]. First, all antecedents have been summarised and categorised; then, the directions of their relationships with UNC were also summarised as increasing, decreasing, or not influencing the UNC occurrence by considering the study design and the data extracted. Researchers worked independently and then agreed upon the findings. The entire research team (see authors) reached consensus about the identified categories and the overall findings in two online meetings.

### Quality appraisal

The 58 studies were evaluated for their methodological quality with the Joanna Briggs Institute Critical Appraisal approach. This was selected according to its capacity to guide the development of high-quality systematic reviews addressing policy and practice interventions [26]. First, different tools were selected according to the design of the studies included, namely the critical appraisal tool for analytical cross-sectional, cohort, and quasi-experimental studies [26–28]. Then, the evaluators were trained in the use of each tool with an online meeting; during the training, multiple exercises were offered to answer each item included in the tools (Y: yes; N: no; U: unclear; NA: not applicable) in order to ensure rigor in the assessment. Then, there were identified three couples of researchers responsible for around 19 studies/each: the evaluation was performed by one researcher and then cross-checked by a second researcher. In the case of disagreements, the entire research team was involved in multiple meetings, in order to discuss the evaluation and reach a consensus. All 58 studies demonstrated sufficient quality and, therefore, all were included in the review.

## Results

### Characteristic of studies

The 58 studies included (Additional File 1) collected from 2006 [29, 30] to 2018–19 [31]. The majority were cross-sectional, except for three cohort studies [30, 32, 33] and one quasi-experimental study [34]. Most of the studies aimed to investigate the occurrence of UNC and its antecedents and only one was performed to validate the MISSCARE survey [35].

Twenty-one studies were conducted in the United States [29, 34, 36–54]; five in Australia [11, 55–58]; three each in Switzerland [59–61] and Cyprus [8, 62, 63]; and two each in England [32, 64], South Korea [65, 66], Israel [67, 68] and China [69, 70]. The remaining were conducted in Brazil, Canada, Denmark, Germany, Korea, Kuwait, Iceland, Italy, Ireland, Mexico, the Philippines, South Africa, Sweden, and Jordan. Only four multi-country studies have been conducted to date [37, 71–73].

Most of the studies involved acute care hospitals, predominantly medical, surgical, rehabilitation, and intensive care units, and only six studies were conducted in nursing homes [33, 44, 45, 54, 60, 61].

The convenience sampling method was used in 47 studies; the remaining 11 studies [29, 33, 35, 42, 45, 54, 60, 61, 64, 73, 74] used random selection. The assessment of both antecedents and the occurrence of UNC was largely based on nursing staff perceptions. Sample sizes ranged from 71 to 33,659 nurses, mostly including registered nurses (RNs) and nursing assistants, with participation ranging from 8.1% to 100%. The majority of them were female (48.9%–100%), with experience in the role ranging between 5.14 and 16.6 years. Only two studies included patients as a target population [32, 70].

Thirty-four studies used a version of the MISSCARE survey [34–38, 40–45, 47–53, 55–58, 62, 63, 66–68, 73–79] while seven used the Basel Extent of Rationing Nursing Care Assessment tool [8, 59–61, 64, 65, 70]. Only one study used the Perceived Implicit Rationing of Nursing Care survey instrument [39].

Studies were conducted with a variable methodological quality. Failures in reporting confounding factors and the strategies implemented to deal with the confounders identified, were the major deficiency for the cross-sectional studies. Among cohort and the quasi-experimental studies fewer failures have emerged (Supplementary Files 2, 3 and 4).

### The antecedents of unfinished nursing care

Antecedents of the UNC have been categorised at the unit, nurse, and patient levels as summarised in Table 3.

**Table 3:** Map of the UNC antecedences investigated to date in available literature

**Unit level**	- Staff levels, as staff adequacy perceived by nurses, patient-to-nurse ratio and hour-per-patient day
	- Workloads
	- Non-nursing tasks
	- Case mix
	- Shift
	- Overtime
	- Work environment
	- Delivery Care system (team model)
	- Ward, unit
	- Location of the hospital/facility
**Nurse level**	- Age
	- Gender
	- Professional experience
	- Education
	- Absenteeism
	- Part time/full time
	- Professional satisfaction
	- Personal accountability
	- Country of origin
**Patient level**	- Clinical instability

*Abbreviation*: *UNC* Unfinished Nursing Care

### Unit level

As reported in Table 4, staff levels, measured as the adequacy of staff perceived by nurses, the nurse-to-patient ratio, or the hours-per-patient day, have been the most investigated UNC antecedent to date. According to the adopted measure (e.g., adequacy versus inadequacy), studies have documented a positive or negative influence on UNC. Evidence has been accumulated regarding the relationship between a low nurse-to-patient ratio and the increase in the UNC occurrence. Exceptions have been documented by Orique et al. [40] and Zhu et al. [70] in their cross-sectional studies, and by Griffiths et al. [32] underlying a non-linear effect when the nursing hours per patient day were greater than seven. Regarding a component of staff adequacy, as the nursing unit’s number of bedside duty hours out of the number of hours offered by licensed and unlicensed personnel (= skill mix), only Castner et al. [41] documented that an increased skill mix decreased the occurrence of UNC.

**Table 4:** Unfinished nursing care antecedents and the direction of their relationship, according to the study design

Antecedents	Author(s)	Brief description	Study design	Outcome: Unfinished nursing care		
				**↓**	**↑**	**≈**
**Unit level**						
Staffing levels, including staff adequacy as perceived by nurses, patient-to-nurse ratio and hour-per-patient day	Al-Kandari et al., 2009 [80]	More RNs in the unit (some of the tasks)	Cross-sectional	*		
	Ball et al., 2018 [72]	Better nurse staffing (mediation analysis)	Cross-sectional	*		
	Blackman et al., 2018 [56]	Nursing staff perceived as more adequate	Cross-sectional	*		
	Cho et al., 2015 [74]	Working in the highly staffed units (compared with low staffed units)	Cross-sectional	*		
	Kalisch & Lee, 2010 [38]	Respondents who perceived their unit staffing level to be high (compared with staff who felt staffing was inadequate)	Cross-sectional	*		
	Kalisch et al., 2011 [43]	Staff who perceived their staffing as adequate (versus inadequate)	Cross-sectional	*		
	Nelson, 2017 [44]	Better perceptions of staffing adequacy (also for licensed staffing)	Cross-sectional	*		
	Orique et al., 2016 [40]	Better unit staffing adequacy perception	Cross-sectional		*	
	Park et al., 2018 [46]	Higher staffing and resource adequacy score	Cross-sectional	*		
	Schubert et al., 2013 [59]	Better nurse practice environment ‘staff resource adequacy’ at the unit level	Cross-sectional	*		
	Winsett et al., 2016 [50]	Higher staffing adequacy perception reduces reasons for MNC (communication, material resources, labour resources)	Cross-sectional	*		
	Zúñiga et al., 2015 [60]	Higher staffing and resources adequacy	Cross-sectional	*		
	Castner et al., 2014 [41]	Increased skill mix	Cross-sectional	*		
	Duffy et al., 2018 [45]	Higher staffing/resource adequacy as measured with the PES-NWI	Cross-sectional	*		
	Hessels et al., 2015 [29]	PES-NWI subscale: better staffing and resource adequacy	Cross-sectional	*		
	Smith et al., 2018 [49]	Higher staffing and resource adequacy (PES-NWI subscale)	Cross-sectional	*		
	Griffiths et al., 2018 [32]	Higher health care assistant staffing levels (medical wards)	Cohort	*		
	Griffiths et al., 2018 [32]	Higher RN staffing levels (medical wards)	Cohort	*		
	Griffiths et al., 2018 [32]	Higher RN staffing level (wards that care for older people)	Cohort		*	
	Blackman et al., 2019 [11]	Staffing inadequacy as perceived by nurses	Cross-sectional		*	
	Blackman et al., 2019 [11]	Insufficient staff	Cross-sectional		*	
	Blackman et al., 2014 [55]	Issues in nursing care resource provision	Cross-sectional		*	
	Bragadòttir et al., 2016 [76]	Nurses who perceived adequate staffing ≤ 50% of the time (compared with those who felt it was adequate 100% of the time)	Cross-sectional		*	
	Cho et al., 2016 [65]	Low nurse staffing levels	Cross-sectional		*	
	Kalisch et al., 2011 [43]	Nurses who perceived their staffing as less adequate	Cross-sectional		*	
	Al-Kandari et al., 2009 [80]	More patients in the unit (completion of routine Foley catheter care and with oral hygiene)	Cross-sectional		*	
	Al-Kandari et al., 2009 [80]	More patients assigned (completion of routine Foley catheter care, with developing/updating NCP, with dressing changes, and providing comfort talk to the patients)	Cross-sectional		*	
	Ball et al., 2014 [64]	More patients requiring assistance with daily living	Cross-sectional		*	
	Bragadòttir et al., 2016 [76]	More patients taken care of during the last shift	Cross-sectional		*	
	Cho et al., 2016 [65]	An increase of 1 patient/nurse	Cross-sectional		*	
	Drach-Zahavy & Srulovici, 2019 [67]	Higher workload as the patient-to-nurse ratio (also used for path analysis)	Cross-sectional		*	
	Friese et al., 2013 [51]	Higher number of patients cared for during the last shift (oncologic units)	Cross-sectional		*	
	Kalisch et al., 2011 [43]	Nurses who cared for more patients in the previous shift	Cross-sectional		*	
	Orique et al., 2016 [40]	More patients under care	Cross-sectional		*	
	Palese et al., 2015 [77]	Lower daily care in minutes offered by NAs	Cross-sectional		*	
	Schubert et al., 2013 [59]	Higher patient-to-nurse ratio at the unit level (in a separate model)	Cross-sectional		*	
	Schubert et al., 2013 [59]	Higher patient-to-nurse ratio at the unit level (in an adjusted model)	Cross-sectional			*
	VanFosson et al., 2018 [39]	Mean nursing care hours provided by float staff	Cross-sectional		*	
	Zander et al., 2014 [81]	Poor nurse-to-patient ratio	Cross-sectional		*	
	Zander et al., 2014 [81]	Poor nurse-to-NA ratio	Cross-sectional		*	
	Ausserhofer et al., 2014 [71]	Lower patient-to-nurse ratios	Cross-sectional	*		
	Ball et al., 2014 [64]	RNs caring for the fewest patients (6.13 or fewer)	Cross-sectional	*		
	Ball et al., 2016 [82]	Shifts with RN staffing levels < 10 patients/RN (compared with those with ≥ 10 patients/RN)	Cross-sectional	*		
	Ball et al., 2016 [82]	Shifts with RN staffing levels ≤ 6 patients/RN	Cross-sectional	*		
	Ball et al., 2016 [82]	Shifts with RN staffing levels < 4 patients/RN (best ratio)	Cross-sectional	*		
	Kalisch et al., 2011 [36]	More hours per patient day	Cross-sectional	*		
	Kalisch et al., 2011 [36]	More RN hours per patient day	Cross-sectional	*		
	Kalisch et al., 2012 [83]	More hours per patient day	Cross-sectional	*		
	Liu et al., 2018 [69]	Lower day shift patient-to-nurse ratio (or workload)	Cross-sectional	*		
	Nelson, 2017 [44]	More RN hours per resident day rate	Cross-sectional	*		
	Palese et al., 2015 [77]	Fewer patients in their charge during the last shift	Cross-sectional	*		
	Palese et al., 2015 [77]	More daily care offered by RNs (in minutes/day)	Cross-sectional	*		
	Zhu et al., 2019 [70]	Lower nurse-to-patient ratios	Cross-sectional		*	
	Griffiths et al., 2018 [32]	More RN and health-care assistant hours per patient day	Cohort	*		
	Griffiths et al., 2018 [32]	Additional health care assistant hours per patient day	Cohort	*		
	Griffiths et al., 2018 [32]	More RN hours per patient day (high-acuity patients)	Cohort	*		
	Griffiths et al., 2018 [32]	Additional RN hours per patient day	Cohort	*		
	Griffiths et al., 2018 [32]	Increased health care assistant hours per patient day (wards that care for older people)	Cohort	*		
	Griffiths et al., 2018 [32]	More RN hours per patient day (high-acuity patients on early and twilight shifts)	Cohort	*		
	Griffiths et al., 2018 [32]	More RN hours per patient day rate during the previous shift and the subsequent shift (i.e., the early shift)	Cohort	*		
	Griffiths et al., 2018 [32]	More RN hours per patient day	Cohort	*		
	Griffiths et al., 2018 [32]	More health care assistant hours per patient day	Cohort	*		
	Griffiths et al., 2018 [32]	There was no significant main effect for RN hours per patient day	Cohort			*
	Griffiths et al., 2018 [32]	Significant but non-linear association between total care hours per patient day and the rate of missed observations	Cohort	*		
	Griffiths et al., 2018 [32]	Non-linear effects for RN hours par patient days, with incremental benefits continuing at higher staffing levels (> 7 h/day)	Cohort			*
Workloads	Al-Kandari et al., 2009 [80]	Total workloads	Cross-sectional		*	
	Al-Kandari et al., 2009 [80]	More discharges made (back rub/skin care and with oral hygiene)	Cross-sectional		*	
	Al-Kandari et al., 2009 [80]	More transfers made (all nursing tasks)	Cross-sectional		*	
	Al-Kandari et al., 2009 [80]	Performing extraordinary life support	Cross-sectional		*	
	Blackman et al., 2014 [55]	Higher work intensity	Cross-sectional		*	
	Blackman et al., 2014 [55]	Workload unpredictability	Cross-sectional		*	
	Blackman et al., 2017 [73]	Missed lower priority nursing care	Cross-sectional		*	
	Blackman et al., 2017 [73]	Missed higher priority nursing care	Cross-sectional		*	
	Castner et al., 2014 [41]	Increased unit workload	Cross-sectional		*	
	McNair et al., 2016 [42]	Spending more time (more minutes per hour) on tasks (activities of daily living, assessment and monitoring, clinical care, communication with patient, communication with care team, documentation)	Cross-sectional			*
	Nelson, 2017 [44]	Higher perception of workloads (also for licensed staffing)	Cross-sectional		*	
	Orique et al., 2016 [40]	Higher unit-level nurse workload (number of admissions, discharges, transfers in, and transfers out)	Cross-sectional			*
	Griffiths et al., 2018 [32]	More admissions per RN	Cohort		*	
	McNair et al., 2016 [42]	Spending less time on documentation (fewer minutes per hour)	Cross-sectional			*
	Srulovici et al., 2017 [68]	Lower workloads, captured as fewer patients per nurse (focal and incoming nurse)	Cross-sectional	*		
Non-nursing tasks	Al-Kandari et al., 2009 [80]	More non-nursing tasks	Cross-sectional		*	
	Bekker et al., 2015 [84]	High occurrence of non-nursing tasks (‘Delivering and retrieving food trays’)	Cross-sectional		*	
	Bekker et al., 2015 [84]	High occurrence of non-nursing tasks (‘Routine phlebotomy/blood drawing for tests’)	Cross-sectional		*	
	Bekker et al., 2015 [84]	High occurrence of non-nursing tasks (‘Cleaning patients’ rooms and equipment’)	Cross-sectional		*	
	Liu et al., 2018 [69]	Fewer non-professional tasks	Cross-sectional	*		
Case mix	Kalisch et al., 2011 [36]	Case mix index	Cross-sectional			*
Shift	Blackman et al., 2014 [55]	Shift time (antemeridian versus post)	Cross-sectional		*	
	Blackman et al., 2018 [56]	Morning shifts (compared with afternoon shifts)	Cross-sectional	*		
	Kalisch et al., 2011 [43]	Working day shifts (compared with night shifts)	Cross-sectional		*	
	Kalisch et al., 2011 [43]	Night shift workers (compared with day shift workers)	Cross-sectional	*		
	Kalisch et al., 2013 [37]	RNs who worked night shifts (compared with day shifts)	Cross-sectional	*		
	Knopp-Sihota et al., 2015 [31]	Most frequently worked evening and night shifts versus morning shifts	Cohort		*	
	Saqer et al., 2018 [78]	Nurses working in mixed (day and night) shift schemes	Cross-sectional		*	
Overtime	Blackman et al., 2019 [11]	Undertake extra shifts (from never to up to 20)	Cross-sectional		*	
	Chapman et al., 2016 [57]	Nurses working overtime for 5–12 h and > 12 h (compared with staff who did not work any overtime hours)	Cross-sectional		*	
	Cho et al., 2016 [65]	Overtime (RNs worked beyond the contracted hours)	Cross-sectional		*	
	Nelson, 2017 [44]	Working > 12 h of overtime (also for licensed staffing)	Cross-sectional		*	
	Phelan et al., 2018 [75]	Nurses who worked more than 39 h a week (correlation with educational nursing duties)	Cross-sectional	*		
Work environment	Blackman et al., 2019 [11]	Dissatisfied working as a team	Cross-sectional		*	
	Bragadòttir et al., 2016 [76]	Better nursing teamwork	Cross-sectional	*		
	Bragadòttir et al., 2016 [76]	Increased teamwork	Cross-sectional	*		
	Chapman et al., 2016 [57]	Higher teamwork score (Nursing Teamwork Survey)	Cross-sectional	*		
	Kalisch & Lee, 2010 [38]	Higher teamwork overall scores	Cross-sectional	*		
	Nelson, 2017 [44]	Better nursing teamwork (also for licensed staffing)	Cross-sectional	*		
	Zúñiga et al., 2015 [60]	Higher teamwork and safety climate (correlated to rationing in the subscales activities of daily living and caring, rehabilitation, and monitoring	Cross-sectional	*		
	Kalisch & Lee, 2010 [38]	Nursing Teamwork Survey subscale: higher trust	Cross-sectional	*		
	Kalisch & Lee, 2010 [38]	Nursing Teamwork Survey subscale: higher team orientation	Cross-sectional	*		
	Kalisch & Lee, 2010 [38]	Nursing Teamwork Survey subscale: higher backup behaviour	Cross-sectional	*		
	Kalisch & Lee, 2010 [38]	Nursing Teamwork Survey subscale: higher sharing of mental model	Cross-sectional	*		
	Kalisch & Lee, 2010 [38]	Nursing Teamwork Survey subscale: better team leadership	Cross-sectional	*		
	Ausserhofer et al., 2014 [71]	More favourable work environments	Cross-sectional	*		
	Ball et al., 2014 [64]	Better practice environment	Cross-sectional	*		
	Duffy et al., 2018 [45]	Positively rated work environment	Cross-sectional	*		
	Kim et al., 2018 [66]	Better nursing work environment	Cross-sectional	*		
	Kim et al., 2018 [66]	Higher nursing work environment subscale scores (nurse participation in hospital affairs; nursing foundations for quality of care; nurse manager ability, leadership, and support of nurses; staffing and resource adequacy; collegial nurse–physician relations)	Cross-sectional	*		
	Liu et al., 2018 [69]	Better work environment	Cross-sectional	*		
	Papastavrou et al., 2014 [8]	Higher rating of Revised Professional Practice Environment subscales: Internal Work Motivation, Leadership and Autonomy, Staff Relations with Physicians, Teamwork and Communication About Patients	Cross-sectional	*		
	Park et al., 2018 [46]	Good environment units (compared with poor environment units)	Cross-sectional	*		
	Smith et al., 2018 [49]	A one standard deviation increases in the nurse work environment	Cross-sectional	*		
	Smith et al., 2018 [49]	Better nurse work environment and higher collective efficacy	Cross-sectional	*		
	Hessels et al., 2015 [29]	Higher PES-NWI composite score	Cross-sectional	*		
	Hessels et al., 2015 [29]	Higher score on each of the five dimensions of the practice environment of PES-NWI	Cross-sectional	*		
	Smith et al., 2018 [49]	Higher PES-NWI composite score	Cross-sectional	*		
	Blackman et al., 2014 [55]	More communication issues	Cross-sectional		*	
	Castner et al., 2014 [41]	More RN communication problems	Cross-sectional		*	
	Palese et al., 2015 [77]	Communication tensions between RNs and NAs	Cross-sectional		*	
	Duffy et al., 2018 [45]	Better collegial relationships as measured with the PES-NWI	Cross-sectional	*		
	Hessels et al., 2015 [29]	PES-NWI subscale: better collegial nurse physician relationships	Cross-sectional	*		
	Park et al., 2018 [46]	Higher nurse–physician relations score	Cross-sectional	*		
	Vryonides et al., 2016 [62]	Better instrumental ethical climate score	Cross-sectional		*	
	Vryonides et al., 2016 [62]	Better independence ethical climate score	Cross-sectional		*	
	Vryonides et al., 2016 [62]	Better caring ethical climate score	Cross-sectional	*		
	Vryonides et al., 2016 [62]	Better rules ethical climate score	Cross-sectional	*		
	Vryonides et al., 2016 [62]	Better law and code ethical climate score	Cross-sectional	*		
	Coleman, 2018 [47]	Higher nursing incivility scores	Cross-sectional		*	
	Coleman, 2018 [47]	Higher supervisor total nursing incivility score	Cross-sectional		*	
	Coleman, 2018 [47]	Higher patient/family/visitor’s incivility scores	Cross-sectional		*	
	Coleman, 2018 [47]	Higher workplace incivility	Cross-sectional		*	
	Menard, 2014 [52]	Higher nursing incivility score	Cross-sectional		*	
	Menard, 2014 [52]	Higher supervisor total nursing incivility score	Cross-sectional		*	
	Menard, 2014 [52]	Higher workplace incivility	Cross-sectional		*	
	Menard, 2014 [52]	Higher patient/family/visitor scores (PES-NWI)	Cross-sectional		*	
	Duffy et al., 2018 [45]	Better foundations for quality as measured with the PES-NWI	Cross-sectional	*		
	Hessels et al., 2015 [29]	PES-NWI subscale: higher nursing foundations for quality of care	Cross-sectional	*		
	Duffy et al., 2018 [45]	Better nurse participation as measured with the PES-NWI	Cross-sectional	*		
	Hessels et al., 2015 [29]	PES-NWI subscale: higher nurse participation in hospital affairs	Cross-sectional	*		
	Duffy et al., 2018 [45]	Better leadership and support as measured with the PES-NWI	Cross-sectional	*		
	Hessels et al., 2015 [29]	PES-NWI subscale: better nurse manager leadership, higher ability, higher support of nurses	Cross-sectional	*		
	Bekker et al., 2015 [84]	More independence at work	Cross-sectional	*		
	Castner et al., 2014 [41]	More RN supply problems	Cross-sectional		*	
	Piscotty et al., 2014 [53]	Higher nursing care reminders usage	Cross-sectional	*		
	Piscotty et al., 2014 [53]	Higher scores on the Impact of Healthcare Information Technology Scale	Cross-sectional	*		
	Smith et al., 2018 [49]	A one standard deviation increases in collective efficacy	Cross-sectional	*		
	White et al., 2019 [54]	Higher burnout among RNs	Cross-sectional		*	
	Ball et al., 2014 [64]	Better nurse perception of the quality of nursing care	Cross-sectional	*		
	Labrague et al., 2019 [31]	Higher scores on the Caring Behaviour Inventory	Cross-sectional	*		
	Ball et al., 2014 [64]	Better nurses overall grading of patient safety on their unit/ward	Cross-sectional	*		
	Kim et al., 2018 [66]	Better patient safety culture	Cross-sectional	*		
	Schubert et al., 2013 [59]	A more favourably estimated ‘patient safety climate’ at the hospital level	Cross-sectional	*		
	Castner et al., 2014 [41]	More RN errors of commission	Cross-sectional	*		
	Zúñiga et al., 2015 [60]	Higher teamwork and safety climate (correlated to rationing in the subscales activities of daily living and caring, rehabilitation, and monitoring)	Cross-sectional	*		
Delivery Care System	Saqer et al., 2018 [78]	Team nursing vs total patient care	Cross-sectional		*	
Ward, unit	Bragadòttir et al., 2016 [76]	Medical and surgical units (compared with ICUs)	Cross-sectional		*	
	Coleman, 2018 [47]	Medical/surgical units versus emergency department, surgical operating room, and obstetrics	Cross-sectional		*	
	Papastavrou et al., 2014 [8]	Surgical departments (compared with medical wards)	Cross-sectional	*		
	Castner et al., 2014 [41]	Critical care units (compared with other units)	Cross-sectional	*		
	Kalisch et al., 2013 [37]	RNs who worked in rehabilitation (versus ICU)	Cross-sectional		*	
	Hernández-Cruz et al., 2017 [79]	Inpatient service (compared with the emergency department)	Cross-sectional		*	
	Blackman et al., 2019 [11]	Type of residence (e.g., low care, dementia only)	Cross-sectional		*	
Location of the hospital/ facility	Blackman et al., 2014 [11]	Metropolitan work site (versus rural)	Cross-sectional		*	
	Knopp-Sihota et al., 2015 [33]	The location of the facility (urban versus rural)	Cohort		*	
	Knopp-Sihota et al., 2015 [33]	Health care aides: urban versus rural	Cohort		*	
	Blackman et al., 2018 [56]	Region of work (e.g., comparison among Australian areas)	Cross-sectional	*		
	Knopp-Sihota et al., 2015 [33]	Working in a given province	Cohort	*		
	Knopp-Sihota et al., 2015 [33]	Health care aides: province (e.g., Saskatchewan versus others)	Cohort		*	
	Kalisch & Lee, 2012 [48]	Magnet unit staff (compared with non-Magnet hospitals)	Cross-sectional	*		
	Blackman et al., 2019 [11]	Size of the residence (e.g., beds)	Cross-sectional		*	
	Knopp-Sihota et al., 2015 [33]	Beds (small [up to 79] versus medium [up to 120] versus large [< 120])	Cohort	*		
	Knopp-Sihota et al., 2015 [33]	Health care aides: small nursing homes	Cohort		*	
	Blackman et al., 2019 [11]	Residence owner (e.g., private)	Cross-sectional		*	
	Knopp-Sihota et al., 2015 [33]	Not for profit (versus profit)	Cohort	*		
	Nelson, 2017 [44]	Higher bed occupancy rate	Cross-sectional		*	
	Knopp-Sihota et al., 2015 [33]	The organisational context (lower context versus higher context)	Cohort		*	
	Knopp-Sihota et al., 2015 [33]	Health care aides who work on a unit with a lower organisational context	Cohort		*	
**Nurse level**						
Age	Al-Kandari et al., 2009 [80]	Increased age of nurses	Cross-sectional	*		
	Higgs et al., 2016 [58]	Medical care nurses aged > 50 years	Cross-sectional		*	
	Kalisch et al., 2011 [43]	Older nurses	Cross-sectional		*	
	Palese et al., 2015 [77]	Older nursing staff	Cross-sectional	*		
	Phelan et al., 2018 [75]	35–44-year-old age bracket (compared with the 25–34-year-old bracket)	Cross-sectional		*	
	Saqer et al., 2018 [78]	Increased age (regarding the perceived level of MNC)	Cross-sectional	*		
	Knopp-Sihota et al., 2015 [33]	Increased age	Cohort		*	
	Phelan et al., 2018 [75]	Younger community nurses	Cross-sectional		*	
	Knopp-Sihota et al., 2015 [33]	Younger health care aides	Cohort		*	
	Phelan et al., 2018 [75]	Community nurses aged 35–44 years (compared with those aged 25–34 and 55–64 years)	Cross-sectional		*	
	VanFosson et al., 2018 [39]	Between-nurse factors (compared with within-nurse factors)	Cross-sectional		*	
Gender	Ausserhofer et al., 2014 [71]	Female nurses	Cross-sectional	*		
	Kalisch et al., 2011 [43]	Female nurses	Cross-sectional		*	
	Saqer et al., 2018 [78]	Female gender	Cross-sectional	*		
	Chapman et al., 2016 [57]	Male nurses (versus female nurses)	Cross-sectional	*		
	Drach-Zahavy & Srulovici, 2019 [67]	In the path analysis, MNC has emerged as directly influenced by gender	Cross-sectional		*	
	Drach-Zahavy & Srulovici, 2019 [67]	Significant correlations between MNC and gender (p = 0.05)	Cross-sectional		*	
	Papastavrou et al., 2016 [85]	Staff gender	Cross-sectional			*
Professional experience	Ausserhofer et al., 2014 [71]	Nurses with more professional experience	Cross-sectional	*		
	Castner et al., 2014 [41]	More RN experience	Cross-sectional		*	
	Kalisch & Lee, 2010 [38]	Staff with 5–10 years of experience and those with > 10 years of experience (compared with those with ≤ 6 months experience)	Cross-sectional		*	
	Kalisch et al., 2011 [36]	Experience > 5 years	Cross-sectional			*
	Kalisch et al., 2011 [43]	Experienced nurses	Cross-sectional		*	
	Kalisch et al., 2013 [37]	RNs who had ≥ 2 years of role experience (compared with ≤ 6 months)	Cross-sectional		*	
	Kim et al., 2018 [66]	Greater clinical experience	Cross-sectional	*		
	Blackman et al., 2017 [73]	Less clinical experience	Cross-sectional		*	
	Chapman et al., 2016 [57]	Staff with ≤ 6 months of experience (compared with ≥ 10 years)	Cross-sectional	*		
	Kalisch et al., 2011 [43]	Staff with fewer years of experience	Cross-sectional	*		
	Palese et al., 2015 [77]	Lower experience in the medical unit	Cross-sectional		*	
	Phelan et al., 2018 [75]	Community nurses with < 5 years of experience (correlation with e.g., initial client needs assessments, follow-up visits after a re-assessment, liaising with other professionals)	Cross-sectional		*	
Education	Blackman et al., 2019 [11]	Role in residents’ care (RNs versus PNs)	Cross-sectional		*	
	Bragadòttir et al., 2016 [76]	RNs (versus PNs)	Cross-sectional		*	
	Higgs et al., 2016 [58]	Critical care nurses who had worked for a longer time as a RN	Cross-sectional		*	
	Kalisch et al., 2011 [43]	RNs (versus NAs)	Cross-sectional		*	
	Orique et al., 2016 [40]	More advanced job title (RNs versus NAs)	Cross-sectional	*		
	Chapman et al., 2016 [57]	Enrolled nurses (compared with RNs)	Cross-sectional	*		
	Friese et al., 2013 [51]	Nursing assistant as a job title in oncologic units	Cross-sectional	*		
	Kalisch & Lee, 2010 [38]	NAs (compared with nurses)	Cross-sectional	*		
	Kalisch et al., 2011 [43]	NAs (versus RNs)	Cross-sectional	*		
	Griffiths et al., 2018 [32]	Effect of health care assistant staff is stronger (regarding RN staffing)	Cohort	*		
	Blackman et al., 2018 [56]	Region of qualification (e.g., comparison among Australian areas)	Cross-sectional	*		
	Bekker et al., 2015 [84]	More educational opportunities	Cross-sectional	*		
	Kalisch et al., 2013 [34]	Receiving education with didactic presentations, scenarios including role playing (simulation), debriefing, and discussion	Quasi-experimental	*		
Absenteeism	Kalisch et al., 2011 [43]	Those who missed more shifts in the past 3 months (compared with those who did not miss any shifts)	Cross-sectional		*	
	Kalisch et al., 2011 [43]	Nursing staff who missed ≥ 2 shifts in the past 3 months (compared with those who did not miss any shifts)	Cross-sectional		*	
	Kalisch et al., 2011 [36]	Absenteeism	Cross-sectional			*
	Kalisch et al., 2013 [37]	RNs who missed any workdays (compared with those who did not miss any)	Cross-sectional		*	
Part time or full time	Ausserhofer et al., 2014 [71]	Part-time nurses	Cross-sectional	*		
	Phelan et al., 2018 [75]	Community nurses working less than 39 h a week (correlation with child health promotion)	Cross-sectional	*		
	Palese et al., 2015 [77]	Working in a full-time position	Cross-sectional		*	
	Srulovici et al., 2017 [68]	Employment status (full-time versus part-time)	Cross-sectional	*		
Professional satisfaction	Bekker et al., 2015 [84]	Greater satisfaction with current job	Cross-sectional	*		
	Orique et al., 2016 [40]	Greater satisfaction with current position	Cross-sectional	*		
	Siqueira et al., 2017 [35]	Greater satisfaction with position/role	Cross-sectional	*		
	Siqueira et al., 2017 [35]	Greater satisfaction with teamwork	Cross-sectional	*		
	Siqueira et al., 2017 [35]	Greater satisfaction with profession	Cross-sectional	*		
	Knopp-Sihota et al., 2015 [33]	Greater satisfaction in their career	Cohort		*	
	Blackman et al., 2014 [55]	Greater dissatisfaction in current job	Cross-sectional		*	
	Papastavrou et al., 2016 [85]	Less job satisfaction	Cross-sectional		*	
	White et al., 2019 [54]	Greater job dissatisfaction among RNs	Cross-sectional		*	
	Knopp-Sihota et al., 2015 [33]	Job satisfaction (no versus yes)	Cohort		*	
	Knopp-Sihota et al., 2015 [33]	Health care aides less satisfied with their job	Cohort		*	
	Blackman et al., 2014 [55]	Higher intention to leave	Cross-sectional		*	
	Nelson, 2017 [44]	Plans to leave (also for licensed staffing)	Cross-sectional		*	
	Hogh et al., 2018 [30]	Copenhagen Psychosocial questionnaire: higher exposure to bullying (time 1)	Cohort		*	
	Zander et al., 2014 [81]	Higher degree of emotional exhaustion	Cross-sectional		*	
	Knopp-Sihota et al., 2015 [33]	Higher Maslach Burn Out Inventory scores	Cohort		*	
	Knopp-Sihota et al., 2015 [33]	Health care aides who report higher levels of exhaustion and cynicism	Cohort		*	
	Hogh et al., 2018 [30]	Copenhagen Psychosocial questionnaire: Affective organisational commitment	Cohort			*
	Zúñiga et al., 2015 [60]	Greater work stress due to workloads	Cross-sectional		*	
	Zúñiga et al., 2015 [60]	Greater work stress due to conflict and lack of recognition	Cross-sectional		*	
	Zúñiga et al., 2015 [60]	Greater work stress due to lack of preparation	Cross-sectional	*		
	Dhaini et al., 2017 [61]	Physical and mental health factors (presence of joint pain, tiredness, headache)	Cross-sectional		*	
	Knopp-Sihota et al., 2015 [33]	Higher Short Form-8 Physical Health scores	Cohort		*	
	Knopp-Sihota et al., 2015 [33]	Higher Short Form-8 Mental Health	Cohort		*	
	Knopp-Sihota et al., 2015 [33]	Health care aides who have lower efficacy and worse self-reported physical and mental health	Cohort		*	
	Drach-Zahavy & Srulovici, 2019 [67]	Higher conscientiousness	Cross-sectional	*		
	Drach-Zahavy & Srulovici, 2019 [67]	Higher agreeableness	Cross-sectional	*		
	Drach-Zahavy & Srulovici, 2019 [67]	Higher neuroticism	Cross-sectional		*	
	Smith et al., 2018 [49]	Higher scores on the Collective Efficacy Beliefs Scale index	Cross-sectional	*		
Personal accountability	Drach-Zahavy & Srulovici, 2019 [67]	Higher personal accountability	Cross-sectional	*		
	Srulovici et al., 2017 [67]	Higher personal and ward accountability (focal and incoming nurse)	Cross-sectional	*		
Country of origin	Blackman et al., 2017 [73]	Nurses’ country of origin (Australia versus Italy)	Cross-sectional		*	
**Patient level**						
Clinical instability	Al-Kandari et al., 2009 [80]	More unstable patients assigned (adequate documentation)	Cross-sectional		*	
	Al-Kandari et al., 2009 [80]	Higher patient death rate	Cross-sectional		*	
	Ball et al., 2014 [64]	More patients requiring frequent monitoring	Cross-sectional		*	

*Abbreviations*: *ICU* Intensive care unit, *MNC* Missed nursing care, *NA* Nursing assistant, *NCP* Nursing care plans, *PES-NWI* Practice Environment Scale-Nursing Work Index, *PN* Practical nurse, *RN* Registered nurse, ↓ = decrease, ↑ = increase, ≈ no significant findings, no associations/correlations, no clear conclusions

The workloads measured with different metrics as the number of patients admitted or discharged [32, 80] have been reported to increase the occurrence of the UNC when measured as a global score by using the MISSCARE survey [41]. Exceptions have been reported by McNair et al. [42] and Orique et al. [40] where workloads were not found to affect the occurrence of UNC. Moreover, performing non-nursing tasks, which might contribute to increased workloads, has also been reported to affect, mainly increasing, the occurrence of UNC [34, 69, 84].

Regarding working shifts, studies have documented conflicting findings, with some reporting that nurses working during the day shift perceived an increase in the occurrence of the UNC [37, 43, 55] while others did not [33, 56]. Moreover, working overtime has also been documented to increase the perceived occurrence of UNC [11, 44, 57, 65], except for the study performed by Phelan et al. [75].

Differently, concerning the quality of the work environment, including aspects ranging from better communication, better grading of caring ethical climate or patient safety, studies have consistently documented that a better environment decreases the occurrence of UNC [8, 11, 29, 31, 38, 41, 44–47, 49, 52–54, 57, 59, 60, 64, 66, 69, 71, 76, 77, 84]. Effective teamwork [11, 29, 45, 46, 57, 76] as well as a higher score in all dimensions of the Nursing Teamwork Scale (including for example, team leadership, team orientation) [38] were also reported to decrease the perception of UNC. Conversely, communication issues were documented to increase UNC [41, 55, 77]. Moreover, working according to team nurses’ model care delivery as compared to total patient care have been reported to increase the UNC perceptions among nurses [78]. Conflicting findings have emerged instead regarding the type of unit, with Bragadóttir et al. [76] and Coleman [47], documenting that medical and surgical nurses perceived a higher occurrence of UNC compared with those working in other units such as intensive care units. On the other hand, Papastavrou et al. [8] reported that nurses in surgical units perceived low UNC when compared with those working in medical units.

Studies about the influence of the facility/hospital were also performed. Knopp-Sihota et al. [33] and Blackman et al. [55] documented that nurses working in urban and metropolitan hospitals reported a higher occurrence of UNC compared with those working in private hospitals [11]. By contrast, Knopp-Sihota et al. [33] reported that nurses working in a not-for-profit hospital perceived a low occurrence of UNC, similar to that documented in Magnet hospitals [48].

### Nurse level

As reported in Table 4, the age of nurses has been investigated for its role in the perception of UNC with conflicting findings. Some have documented that older age is associated with low levels of UNC [77, 78, 80], while others have reported the opposite [33, 43, 58, 75]. Similarly, professional experience has been documented to have a variable influence on UNC, with more clinical experience associated with a higher perceived occurrence of UNC [37, 38, 41, 43, 57] and others documenting the opposite findings [66, 71, 73, 75, 77]. In addition, also the gender has been investigated with conflicting findings, with some studies reporting that female nurses perceived more UNC [43] or less [71, 78] as compared with male nurses.

Regarding nursing education, RNs were reported as perceiving more UNC compared with other roles [38, 43, 57, 58, 76]. However, some authors [34, 40, 84] reported that higher education as attending multiple educational opportunities, prevents the perception of UNC.

In terms of working profiles, Blackman et al. [11] and Chapman et al. [57] have both documented that working part-time decreases the perception of UNC among nurses while working full time remains unclear [68, 77]. Moreover, nurses reporting a higher ratio of absenteeism have been documented to perceive higher levels of UNC [37, 43], except for Kalisch et al. [36] who did not find any relationship.

Almost all studies, except for the one conducted by Knopp-Sihota et al. [33], have reported similar findings regarding nurses’ dissatisfaction, stress, emotional exhaustion, intention to leave, and other elements of poor professional well-being, all of which increase the perceived occurrence of UNC [35, 40, 84]. Moreover, Drach-Zahavy and Srulovici [67] and Srulovici and Drach-Zahavy [68] documented that nurse’s personal degree of accountability influences their perception of UNC, while Blackman et al. [73] analysed the association between UNC and the country of origin of nurses, documenting that Australian nurses perceived more UNC compared with Italian nurses.

### Patient level

Only two studies have investigated antecedents at the patient levels suggesting that clinical instability may play a role regarding the UNC. Caring for more unstable or critical patients, requiring frequent monitoring or units with higher patient death rate [64, 80] were reported as factors increasing the occurrence of UNC (Table 4).

## Discussion

### Characteristics of studies available

Several studies were conducted in a short period, mainly in the United States (US). Subsequently, the process of missed nursing care concept development firstly reported among US studies, was researched also across the world, especially to Europe [2]. An important impetus for this development has been a project funded by the European Union in 2016, the RANCARE [86], which brought together scholars and practitioners from 34 countries who had worked for four years, giving an international perspective to a relatively unacknowledged nursing problem. However, there are only a few examples of studies conducted at a multi-country level [37, 71–73], where antecedents might function differently according to variances in national health services, education systems, cultures, and resources devoted to the nursing care. Moreover, studies have been mainly focused on the hospital/acute care setting, suggesting that more research is needed in community and nursing home settings to accumulate evidence in these settings of care.

To date, both antecedents and the occurrence of UNC have been studied mainly from the point of view of nursing staff as a self-assessment, perceptions that can be influenced by several biases. Moreover, some studies have examined perceptions of both nurses and nurse’s aides [40, 43, 48, 77]; these professionals have a different scope of practice, and this may have influenced their assessments. Only two studies involved patients, a perspective that should be considered in future research to better understand the occurrence of UNC also from their point of view [87].

The study designs were largely cross-sectional in nature, with mainly convenience samples and a great variance in the participation rates, that all might have introduced biases in the evaluation of both antecedents and the UNC occurrence. In addition, antecedents and UNC occurrence have been largely measured at the same time point, thus assuming that the former has influenced the latter whereas control variables and/or confounding factors (e.g., the overtime, as paid or not) were not investigated. These issues have been reported also by Griffiths et al. [88] regarding the state of the art of the evidence about the nursing staffing and outcomes.

Longitudinal, pre- and post-study designs, or comparative studies are encouraged to increase the strength of evidence, by quantifying also the benefits of reducing/minimizing unfinished care and the costs, feasibility and long-term sustainability of implemented interventions. However, study designs should be considered in light of the complexity of the nursing care and the issue under study: UNC occurs in the real world of nursing across the world as a multifactorial phenomenon. Assessing precise antecedents might be difficult—moreover, designing interventional studies manipulating for example, the work environment, or the number of staff might be not feasible given the complexity of the turbulence of environments, and the challenges of the long-term implementation. Therefore, an in-depth discussion regarding the research issues in this field is required, analogously to that already developed in the context of nursing staffing and outcomes [88].

### The antecedents of unfinished nursing care

Conceptual articles have highlighted that UNC is influenced by patient care demand, resource allocation, and relationship/communication issues [14] as well as by patient, organisational, nursing work environment, philosophy of care, and nurse variables [3]. In recent years, there has been a more comprehensive consideration of macro-, meso-, and micro-level factors by examining how upper-level management might affect clinical nurses and, consequently, UNC at the bedside [16]. However, according to the findings of this review, primary studies available to date appear to have investigated antecedents only at the unit, nurse, and patient levels. Therefore, despite a clear conceptualisation of the importance of the factors at the system level [16], empirical studies seem to have captured only a limited extent of factors with heterogeneous findings.

At the unit level, the staff adequacy as measured with different methods (e.g., workloads [40, 41, 44, 55, 68, 80] versus nurse-to-patient ratio [43, 51, 59, 65, 67, 69–71, 81, 82], using subjective or objective data) influences the occurrence of UNC. Moreover, other processes such as patients’ admissions and discharges or caring for patients with complex needs increase the occurrence of UNC, likely because they affect workloads in an unpredictable manner that requires a revision of staff dynamics and resource assignments [89]. On the other hand, performing non-nursing tasks [69, 80, 84] were documented to increase UNC as well as working overtime [11, 44, 57, 65]. Unfinished care might be triggered by the underuse of nurses, constrained to compensate for deficiencies in auxiliary resources thus leaving nursing care undone; conversely, unfinished care might be the consequence of the tiredness and reduced performance of nurses due to the amount of overtime work. A clear direction has not emerged regarding shifts (e.g., morning versus nights) and this might be due to the different patterns of both shifts (e.g., 12 h) and workloads established at the unit level. Specifically, those working morning shifts are required to deal with the high number of concentrated activities, while those working night shifts have few resources to meet care needs.

Within the unit level, the findings mainly reflect the structural variables [90] of the unit, with modifiable factors that might reduce or minimise UNC. These factors include adequate staff levels, preventing nurses from performing non-nursing tasks and working overtime, and implementing strategies to deal with the unpredictability of workloads for some shifts. The findings support the conceptualisation of Jones et al. [16] that factors affecting the occurrence of UNC can be considered in light of micro-economic theories as the efficient allocation of scarce resources to nursing care.

Several studies [8, 11, 29, 31, 38, 41, 44–47, 49, 52–54, 57, 59, 60, 62, 64, 66, 69, 71, 76, 77, 84] have concluded that a better work environment leads to a decrease in the UNC. Hence, promoting greater communication, better caring ethical climate, and respect among nurses and across health care professionals, all reduce or minimise the UNC. These factors, mainly reflecting the process variables of the unit [90], suggest that there is a need to invest in good practice environments for nurses, a strategy that can be developed by nurse managers but requires profound support from the entire system and education to work together effectively. Indeed, the findings that emerged regarding Magnet hospitals [48] and some hospitals/units (rural versus urban [11, 33], surgical versus medical [8, 47, 76]) can explain their capacity to minimise or reduce the UNC as work environments where nursing care is supported and valued.

Studies investigating the relationships between some individual characteristics of nurses (e.g., age, gender, and work experience) and the occurrence of UNC have mainly reported conflicting findings. Some authors also included variables that are not usually measured, such as the nurse’s personality and the country of origin [67, 73] and no trends in this dimension were detected. The interest in individual variables seems to be linked with the fact that the UNC has been investigated mainly as nurses’ perceptions; therefore, it is influenced by the profile of the nurse. However, apart from some antecedents (e.g., education), most of them appear to be unmodifiable, thus suggesting that they should be considered by nurse managers while, for example, they compose shifts that mix different nurse profiles (e.g., age, gender, education). Conceptually, authors have emphasised that nurses’ experience [36–38, 41, 43, 57, 66, 71, 73, 75, 77], education [11, 32, 34, 38, 40, 43, 51, 56–58, 76, 84], and skill mix [41] may influence the quality of the decision-making processes and, ultimately, the occurrence of UNC. However, when nurses are called to make decisions on how to allocate the limited time available, they desire to provide the best care for their patients and eliminate unfinished care. They also need to be supported in making decisions though a positive ethical climate and organisational guidance [62]. Unfortunately, the mental processes involved in decision-making regarding care that can be left undone has been unexplored and more studies are needed to increase understanding of how nurses set priorities while they are trying to cope with the endless needs of patients in the complex environments of the contemporary care facilities [91]. This reflection might also explain why nurses perceive more UNC [38, 43] compared with nurse’s aides suggesting that in studies investigating the unfinished care perceived, a stratification of the responders according to their educational level, is required. Additionally, some of the nurse variables that have been investigated seem to play a dual role as antecedents and as consequences of UNC. For example, decreased professional satisfaction levels [33, 35, 40, 54, 84, 85] might lead to increase the unfinished care but also might be a consequence of the UNC, as reported in conceptual models [15].

Patient characteristics as antecedents of UNC have been poorly investigated. According to the available studies, clinical instability [64, 80] can increase the need for nursing care due to the additional care patients required in context with limited resources. Moreover, only recently the patients’ perceptions about UNC have been summarised [63]: authors concluded that many of the unmet needs perceived by patients do not always correspond to the perceptions of nurses. It is therefore vital to understand the UNC phenomenon from the patients’ point of view with more research; this endeavour will allow researchers to design appropriate interventions. For example, complex clinical cases might require more care with unpredictable flows that can be provided with flexible processes and models of care delivery.

### Limitations and recommendations for research

This systematic review has several limitations. Despite the rigorous approach, some studies might be missed for different reasons and among other, the fact that no quantitative measures (Cohen’s kappa coefficient) were used to evaluate the agreement across researchers regarding the study inclusion. Moreover, according to the limitations applied in the languages (English, Greek, Dutch, and Italian) a potential publication bias might have been introduced. Furthermore, the timeframe was limited by including only studies published after 2004, the year when the first concept pertaining to the UNC was established [4, 21]. However, studies using different key words might have been performed before the establishment of the mentioned conceptual definition; other studies might have been conducted after without using the conceptual definition, leading in both cases to a publication bias. In addition, to map antecedents, the search terms identified were general and designed to capture all studies in the field and not those addressing specific (known) antecedents of the UNC (for example shift patterns) [20]. Finally, there were excluded those studies conducted in specific settings (paediatric, psychiatric) according to the available knowledge [21]: the inclusion of these settings in future reviews might contribute to broaden the evidence available.

In performing the data extraction, some antecedents might have been neglected in favour of providing a comprehensive map of those investigated to date. Commonalities across antecedents by using an inductive approach [25] were searched to summarise the data: although an investigator triangulation was performed [92], researchers might have been influenced in the data analysis process by their previous background and experience regarding the issue. Furthermore, the relationship between antecedents and the UNC as decreasing, increasing or not influencing its occurrence, has not been weighted in its evidence according to the study design or for example the effect size, suggesting an area of improvement in future reviews.

## Conclusion

To the best of our knowledge, this is the first systematic review summarising the antecedents of the unfinished nursing care. Several studies have been conducted throughout the world, indicating a clear interest in this research field. However, the available evidence has mainly been collected with cross-sectional designs, performed at the hospital level, and describing nursing staff perceptions as collected with different tools. Hence, more robust studies are needed in this field challenging the multifactorial nature of the UNC where assessing precise antecedents might be difficult.

Several antecedents of UNC were investigated to date at the unit, nurse, and at the patient levels. At the unit level, (a) structural factors such as an adequate staff levels and strategies to deal with unpredictable variations in the workloads, and (b) process factors, as investing in good work environments for nurses, are highly recommended to minimize/reduce the occurrence of the UNC. At the nurse and patient levels, no clear trends emerged regarding modifiable factors.

The antecedents emerged can be used to design interventional studies in the field that are also aimed at changing the patterns of research from merely descriptive to evaluate the effectiveness of interventions targeting some modifiable factors. This endeavour could minimise and/or reduce the UNC and, ultimately, ameliorate patient, nurse, and system outcomes. Future studies should also consider community settings and involve more robust measures by using different sources of data to identify additional meaningful factors that could contribute to explain the UNC. However, an in-depth discussion regarding the research issues in this field is recommended in order to design studies capable to add value and, therefore, to inform policy-makers shaping nursing services.

## Supplementary Information

**Additional file 1.**

## Data Availability

All data generated or analysed during this study are included in this published article [and its supplementary information files].

## Abbreviations

UNC: Unfinished Nursing Care
SPIDER: Sample, Phenomenon of Interest, Design, Evaluation, Research
PROSPERO: International Prospective Register of Systematic Reviews
CINAHL: Cumulative Index to Nursing and Allied Health Literature
RNs: Registered Nurses
US: United States
