# Supplementary material for: Antecedents of unfinished nursing care: a systematic review of the literature
Source: BMC Nurs. 2022 Jun 14;21:137. doi: 10.1186/s12912-022-00890-6 (PMC9195215; doi:10.1186/s12912-022-00890-6)
Supplement: Supplementary file 1 — Additional file 1. [file 12912_2022_890_MOESM1_ESM.docx]

**Supplementary file 1**. Study characteristics, according to the study design

| **Authors, years, country, setting, and study period** | **Aims and study design** | **Sampling method, target population, participants, and demographics** | **Instrument/tools used to measure explanatory variables and UNC endpoint** |
| --- | --- | --- | --- |
| **Cross-sectional studies** |  |  |  |
| Al-Kandari et al., 2009 [54]  Kuwait  General medical and surgical wards of the regional hospitals (n = 5) of the Ministry of Health  Period: N/A | To assess the workload of nurses; the nursing activities (tasks) nurses commonly performed on medical and surgical wards; elements of nursing care activities left incomplete by nurses during a shift; factors contributing to task incompletion; and the relationship between stafﬁng, demographic variables, and task incompletion  Exploratory study | Convenience  Nurses: 820  Participants: 780 (95%)  Females: 73%  Median age: 29.9 yrs  Nursing certiﬁcate: 4.5%  BSN: 33.8%  Associate degree or equivalent in nursing: 61.6%  Mean experience as nurse: 5.14 yrs | Staff characteristics: age, gender, nationality, area of work, education, years of experience  Unit profile: bed capacity of the unit, nurse-to-patient ratio, number of unstable patients assigned, emergencies encountered during the shift, frequency of various nursing and non-nursing tasks performed during the shift  Nursing activities: tasks left undone modified in the items  Modified tasks left undone instrument |
| Ausserhofer et al., 2014 [46]  Belgium, England, Finland, Germany, Greece, Ireland, the Netherlands, Norway, Poland, Spain, Sweden, Switzerland  European hospitals (n = 488)  Period: 2009–2010 | 1. To describe the prevalence and patterns of nursing care left undone in a large sample of hospitals across 12 European countries  2. To explore the association between the organisational context of nursing – including the nurse work environment, nurse staffing, and requirements that nurses carry out non-nursing tasks – and nursing care left undone  Multi-country, multi-level cross-sectional study | Convenience  Professional nurses: 33,659  Participation: 62%  Females: 93%  BSN: 54%  Average experience: 10.3 yrs | Quality of the nurse work environment: staffing and resource adequacy, nurse manager ability, leadership, support of nurses, collegial nurse–physician relations, nurse participation in hospital affairs, nursing foundations for quality of care  Nurse staffing level  Non-nursing tasks  Staff characteristics: gender, nursing education, employment level, professional experience in the hospital where they were currently working  Potential confounding variables: teaching status, high technology, that is, hospitals providing open heart surgery or organ transplantation, hospital size  RN4CAST nurse questionnaire |
| Ball et al., 2014 [39]  England  General medical/surgical wards (n = 401) in 31 acute National Health Service hospitals  Period: 2010 | To describe the nature and prevalence of care left undone (as reported by nurses) and explore its association with nurse staffing levels and nurse ratings of the quality of care and patient safety environment  Cross-sectional study | Random stratified  Nurses: 2,917  Participation: 39%  Females: 92%  Mean age: 39.6 yrs (SD 10.1 yrs)  BSN: 27%  Length of service as a nurse: 13.8 yrs (SD 10.6 yrs) | Nurse staffing: patients per RN providing direct care, patients per non-RN staff, proportion of the nursing team providing direct care that were RNs  Nurse work environment: managerial support for nursing, nurse participation in hospital affairs, doctor–nurse relations, promotion of care quality  Quality of care  Patient safety  Care left undone: ‘On your most recent shift, which of the following activities were necessary but left undone because you lacked the time to complete them?’; 13 nursing care activities were presented  Practice Environment Scale of the Nursing Work Index  One item from the Agency for Healthcare Research and Quality’s hospital survey  Care left undone |
| Ball et al., 2016 [85]  Sweden  General medical or surgical wards (n = 79) of acute hospitals in Sweden  Period: 2010 | 1. To examine factors associated with RN reports of care left undone on acute medical/surgical wards  2. To describe the relationship between staffing levels and care left undone  Cross-sectional study (part of the RN4Cast study) | Sampling method: N/A  RNs: 33,083  Participation: 70%  Females: 93.1%  Mean age: 39.7 yrs  BSN: 58.8% | Demographics: age, gender, education, working hours, last shift worked, role, length of service  Nurse staffing: patients/RN, patients/nursing support worker, total nurse staffing  Patient dependency and acuity  Practice environment: nurse participation in hospital affairs, managerial support, promotion of care quality, relationships between nurses and doctors  Role in care provision  Transferrable activity  Elements of MNC  Practice Environment Scale of the Nursing Work Index  Basel Extent of Rationing of Nursing Care instrument |
| Ball et al., 2018 [47]  Belgium, England, Finland, Ireland, Netherlands, Norway, Spain, Sweden, Switzerland  Hospitals (n = 300)  Period: 2009–2010 | To examine if MNC mediates the observed association between nurse staffing levels and mortality  Observational study | Convenience  RNs: 26,516  Participation: 62%  N/A | Mortality following surgery within 30 days of admission: administrative data on discharge status, length of stay and adjusted for surgical procedure undergone, patient age, sex, and admission type  Elements of MNC  Demographics: number of staff providing direct patient care, number of patients on their ward on the last shift that they worked, nurse practice environment, nurse education levels  Control variables: hospital bed size, teaching status, technology  Practice Environment Scale  MNC: ‘On your most recent shift, which of the following activities were necessary but left undone because you lacked the time to complete them?’; composed of 13 activities |
| Bekker et al., 2015 [58]  South Africa  Medical and surgical units (n = 60) in six public and private hospitals in six provinces of South Africa  Period: N/A | To investigate the relationship between non-nursing tasks, nursing tasks left undone, and job satisfaction among professional nurses in medical and surgical units in private and public hospitals in South Africa  Cross-sectional study | Convenience  Professional nurses: 2,122 (final sample of 1,166)  Participation: 38.2% for private hospitals and 53.3% for public hospitals  Females: 95.6%  BSN: 14.3% | Job satisfaction: speciﬁc aspects of nurses' job, the level of job satisfaction, namely work schedule ﬂexibility, opportunities for advancement, independence at work, professional status, wages, educational opportunities  Non-nursing tasks: delivering and retrieving food trays, performing non-nursing care, arranging discharge referrals and transportation, routine phlebotomy/blood drawing for tests, transporting of patients within hospital, cleaning patients’ rooms and equipment, filling in for non-nursing services not available on off-hours, answering phones, clerical duties  Nursing tasks left undone: adequate patient surveillance, skin care, oral hygiene, pain management, comfort/talk with patients, educating patients and family, treatments and procedures, administer medications on time, prepare patients and families for discharge, adequately document nursing care, develop or update nursing care plans/care pathways, planning care, frequent changing of patient position  Demographics  RN4CAST paper-based survey, section C for nursing care left undone |
| Blackman et al., 2014 [37]  Australia  Australian Nursing and Midwifery Federation-South and Australian Branch Association  Period: 2012 | 1. To explore which factors inﬂuenced the nursing care reported as being missed by nursing staff  2. To estimate and explain how much variance among different factors can be used to predict why nursing care is likely to be missed  Non-experimental exploratory study | Convenience  Nurses: 289  Participation: 100%  Females: 90%  < 25 yrs: 2%; 25–34 yrs: 12%; 35–44 yrs: 20%; 45–54 yrs: 37%; 55–64 yrs: 27%; > 65 yrs: 2%  < 2 yrs of experience: 13%; 2–5 yrs: 14%; 5–10 yrs: 16%; > 10 yrs: 57% | Reason for MNC  MISSCARE Survey (only Part B) |
| Blackman et al., 2017 [48]  Australia, Cyprus, Italy  Australia: Australian Nursing and Midwifery Federation Association  Cyprus: medical and surgical units of six acute care hospitals  Italy: 12 hospitals  Period: N/A | 1. To identify whether the frequencies and types of MNC differ significantly between countries  2. To understand if the incidence of MNC can be modelled and predicted  Non-experimental, exploratory, quantitative study | Australia: randomised  Cyprus and Italy: convenience  Australia: 7,097 nurses and midwives  Cyprus: 959 nurses  Italy: 467 nurses  Sample: 1,896  Participation:  Cyprus: 81%  Italy: 77.9%  Female: 82%  < 25 yrs: 5%; 25–34 yrs: 47%; 35–44 yrs: 21%; 45–54 yrs: 12%; 55–64 yrs: 13%; > 65 yrs: 2%  < 1 yr of experience: 11%; 1–5 yrs: 40%; 5–10 yrs: 30%; >15 yrs: 19%  Non-university: 19%  BSN: 63%  Above BSN: 18% | Demographics characteristics  Working conditions  Elements of MNC: high priority care (e.g., vital signs assessed as ordered, hand washing), intermediate priority care (e.g., ambulation three times day, as ordered, turning patient every 2 hours), low priority care (e.g., monitoring intake/output, full documentation all necessary data)  MISSCARE survey (Part A and B) |
| Blackman et al., 2018 [60]  Australia  Australian Nursing and Midwifery Federation Association  Period: 2012–2015 | 1. To determine what factors would account for maximum variation in the total MNC score  2. To determine why care omissions occur  Non-experimental, descriptive study | Convenience  Nurses and midwifes: N/A  Participation: 1,195  Certificate/enrolled nurse: 15%  RN diploma: 23%  BSN: 40%  Graduate diploma: 13%  MSN or higher: 9% | Demographic characteristics  Care setting type  Working conditions  Elements of MNC  Reasons for MNC  MISSCARE survey (Part A and B) |
| Blackman et al., 2019 [11]  Australia  Residential aged care (number N/A)  Period: N/A | 1. To identify the types and frequencies of MNC  2. To identify the demographic factors that serve as antecedents or have predictive qualities regarding missed residential aged care  Multivariate approach study | Convenience  Staff components: 3,079  Participation: 2,467 (80.1%)  Care workers: 36%  Enrolled nurses: 26%  RNs: 36%  Nurse practitioners: 2% | Demographics: age, years of experience, qualifications, staffing model used in the facility  Missed residential aged care: care done to minimising residents’ distress, nursing care to maximise the residents’ current health status, care to strengthen the residents’ life potential  Elements of MNC  Australian Aged Care Funding Instrument  MISSCARE survey (Part A and B) |
| Bragadòttir et al., 2016 [64]  Iceland  Medical, surgical, mixed medical and surgical, and intensive care inpatient units (n = 27) in one university hospital, three teaching hospitals, and six small regional hospitals  Period: 2012 | To identify the correlates of hospital, unit, and staff characteristics, and nursing teamwork to MNC in one nationalised health care system  Cross-sectional study | Purposive  Staff: 864  Participants: 69.3%  Females: 98.9%  RNs: 62.6%  Practical nurse: 37.4%  < 34 yrs: 28%; 35–44 yrs: 25.1%; 45–54 yrs: 29.1%; > 55 yrs: 17.8%  < 2 yrs of experience: 13.3%; 2–5 yrs: 15%; 5–10 yrs: 18.9%; > 10 yrs: 52.8% | Unit characteristics  Staff characteristics: gender, age, job title, number of hours worked per week, work hours, experience in role, experience on current unit, overtime, sick days, stafﬁng adequacy, number of patients taken care of on the last shift  Elements of MNC  Teamwork trust, team orientation, backup, shared mental model, team leadership  MISSCARE Survey-Icelandic (only Part A)  Nursing Teamwork Survey-Icelandic |
| Castner et al., 2014 [56]  United States  Direct patient care or unit-level management in one specialty children’s hospital, two suburban community hospitals, and two urban tertiary care hospitals  Period: 2011–2012 | To build and to test a multi-level model on the contextual relationships and interactions of individual RN and nursing unit factors on MNC  Descriptive, cross-sectional study | Convenience  Nurses: 2,509 (the final sample is 553)  Participation: 24.2%  Females: 94.3%  Staff nurse: 96.5%  Administrative: 3.5%  Diploma: 13.0%  Associate degree: 36.9%  BSN: 46.7%  MSN or higher: 3.4% | Elements of MNC  Reasons for MNC  Errors of commission: misinterpreting orders, medication error, violating infection precautions, skill error, delegation/supervision error, wrong chart, assignment error  Demographics  Unit-level variables: case mix index, merger status, medication administration errors and near-misses (bar-coding administration system), incident reports  MISSCARE Survey (Part A and B)  Survey subscale adapted from the Practice and Professional Issues Survey  ADL Omissions |
| Chapman et al., 2016 [61]  Australia  Medical, surgical, ICU, specialist wards including coronary care, emergency department, and rehabilitation units in four hospitals  Period: 2014 | 1. To examine teamwork and MNC in one Australian health network  2. To provide evidence of the ameliorating effect of set nurse-to-patient ratios on teamwork and MNC  Descriptive study | Convenience and consecutive  Nurses: 334  Participation: 89.9%  Females: 89.8%  RNs: 91%  Enrolled nurses: 9%  Mean age: 26–34 yrs  BSN: 40%–70%  > 10 yrs in the same role: 30%–70% | Demographics: hospital, gender, year born, highest education level, country where nursing education occurred, length of time nursing, length of time in current ward/unit/department, description of workplace, work hours, job title, overtime in the last three months, number of missed work days in the last three months, plan to leave current position  Elements of MNC  Reasons for MNC  Teamwork: trust, team orientation, backup, shared mental model and team leadership  MISSCARE Survey (Part A and B)  Nursing Teamwork Survey |
| Cho et al., 2015 [81]  Korea  Highly staffed units (n = 4) and low staffed units (n = 9) of one public hospital  Period: 2013 | To compare MNC between nursing units with high versus low nurse stafﬁng to examine the effects of nurse stafﬁng on MNC  Cross-sectional study | Convenience  Nurses: 115 in high stafﬁng units, 117 in low-stafﬁng units  Participation: in high staffing units, 94.3%; in low staffing units, 88.6%  *High-staffing units versus low-staffing units*  Females: 100% versus 95.7%  Baccalaureate or higher degree: 38.3% versus 32.8%  < 1 year of RN experience: 27.8% versus 17.1% | Elements of MNC  Reasons for MNC  MISSCARE survey (Part A and B) |
| Cho et al., 2016 [40]  South Korea  Acute hospitals (n = 60)  Period: 2008–2009 | To explore the association of nurse staffing and overtime with patient safety, quality of care, and care left undone  Cross-sectional study | Stratified randomisation  Staff: N/A  Participation: 96.2%  Females: 95%  Mean age: 28 yrs (SD 4.8 yrs)  Diploma: 51.5%  Baccalaureate or higher: 48.5%  Mean experience: 5.5 yrs (SD 4.6 yrs) | Demographics: age, gender, highest education, years worked as a nurse, job status, job security, working unit, and last shift worked, hospital characteristics  Patient safety  Quality of care  Care left undone  Nurse staffing level  Nurse’s overtime  Basel Extent of Rationing of Nursing Care  Care left undone  Agency for Healthcare Research and Quality’s Hospital Survey on Patient Safety Culture  Nurses’ reports on the quality of nursing care on their unit |
| Coleman, 2018 [68]  New York, United States  Hospitals (n = 3) in rural western New York  Period: N/A | To investigate a potential relationship between workplace incivility and MNC  Descriptive, cross-sectional study | Convenience  RNs: 478 (the final sample was 102)  Participation: 24.1%  Females: 90.2%  25–34 yrs: 33.3%; 35–44 yrs: 26.5%; 55–64 yrs: 13.7%  Associate degree in nursing: 58.8%  BSN: 34.3%  > 6 months to 2 yrs of experience: 16.7%; 2–5 yrs: 19.6%; 5–10 yrs: 25.5%; > 10 yrs: 37.3% | Elements of MNC  Reasons for MNC  Sources of incivility: inappropriate jokes, hostile climate, free-riding, abusive supervision, gossip/rumours, lack of respect, displaced frustration  MISSCARE Survey (Part A and B)  Nursing Incivility Scale |
| Dhaini et al., 2017 [51]  Switzerland  Swiss nursing homes (n = 162)  Period: 2012–2013 | 1. To assess the prevalence of implicit rationing of direct resident care, including rationing of ADL and of caring, rehabilitation, and monitoring  2. To explore the relationship between care workers’ health and presenteeism regarding implicit rationing of care  Secondary analysis of data from the Swiss Nursing Homes Human Resources Project | Randomised  Care workers: 5,325 (the final sample size was 3,239, with a response rate of 76.6%)  Participation: N/A  Females: 92.2%  RNs: 28.1%  Licensed practical nurse: 24.1%  Certified nursing assistant: 19.1%  Nurse’s aide: 28.8%  ≤ 30 yrs: 21.4%; 31–40 yrs: 18.2%; 41–50 yrs: 27.6%; > 50 yrs: 32.8%  ≤ 5 yrs of experience: 20.6%; 6–10 yrs: 23.1%; 11–15 yrs: 18.7%; 16–20 yrs: 13.2% | Care worker personnel: sociodemographic and professional data, perceptions of health and quality of care  Facility profile questionnaire: nursing home facility characteristics  Questionnaire on physical health factors: self-reported back pain, joint pain, and headache  Self-reported mental health factors: tiredness, sleeplessness, work-related emotional exhaustion  Presenteeism: number of days care workers had attended work in spite of feeling ill and unﬁt  Elements of rationed nursing care  Basel Extent of Rationing of Nursing Care, nursing home version |
| Drach-Zahavy & Srulovici, 2019 [42]  Israel  Internal, surgical, intensive, oncological, operating, psychiatric, paediatric, obstetrics, and emergency units  Period: 2017 | To examine the mediating role of nurses’ personal accountability in the relationships between nurses’ personality and MNC  Multi-centre cross-sectional study | Snowball  RNs: 290  Participation: 100%  Females: 71.3%  Mean age: 38.63 yrs (SD 9.79 yrs)  BSN: 74.2%  Mean nursing seniority: 13.44 yrs (SD 9.79 yrs) | Nurse’s personality: agreeableness, conscientiousness, neuroticism, extraversion, openness to experience  Mediator variable: personal accountability  Control variables: nurse and shift characteristics  Elements of MNC  MISSCARE survey (only Part A)  44-item Big Five Inventory  19-item 3D Accountability Questionnaire |
| Duffy et al., 2018 [65]  United States  One community hospital  Period: 2017 | 1. To describe the phenomenon of MNC and evaluate its associations between selected individual and organisational factors  2. To describe the occurrence and extent of MNC types  3. To examine the relationships between nursing staff characteristics and MNC  4. To examine the relationship between the nursing work environment and MNC  5. To examine the relationship between the combination of nursing staff characteristics and nursing work environment and MNC  Cross-sectional correlational study | Stratified randomly selected  Selected nurses: 201 (138 returned completed surveys)  Participation: 74%  Females: 97.1%  25–34 yrs: 46.4%; 35–44 yrs: 17.4%; 45–54 yrs: 20.3%  BSN: 60.1%  < 6 months of experience in current role: 3.6% | Nurse demographic characteristics  Reasons for MNC  Nurses’ perceptions of the work environment: nurse participation in hospital affairs, nursing foundations for quality care; nurse manager ability, leadership, and support of nurses; staffing and resource adequacy; collegial nurse-physician relations; all had acceptable reliability  MISSCARE survey (only part A)  Practice Environment Scale-Nursing Work Index |
| Friese et al., 2013 [83]  Midwestern United States  Oncological and medical/surgical units (n = 62) of nine hospitals in the Midwestern United States  Period: 2008–2009 | To quantify the degree of MNC in oncology units, compare MNC between oncology and non-oncology medical surgical units, and identify correlates of MNC in oncology units  Secondary analysis | Convenience  Nurses: 2,318  Participation: 59.8%  *Non-oncological versus Oncological units*  Females: 91.1% versus 91.6%  RNs: 73.4% versus 74.1%  26–34 yrs: 31.5% versus 29.9%  35–44 yrs: 24.1% versus 25.1%  Associate degree: 39.7% versus 37.7%  BSN: 40.0% versus 42.0%  < 5 yrs of experience: 52.2% versus 51.4%  5–10 yrs of experience: 18.5% versus 18.6%  > 0 yrs of experience: 29.3% versus 30.0% | Elements of MNC  Reasons for MNC  Nursing staff characteristics: years of experience, gender, role, education  Work schedules: shift, hours worked  Staffing: absenteeism, reported workloads, perceived staffing adequacy  MISSCARE Survey (Part A and B) |
| Hernández-Cruz et al., 2017 [90]  Mexico  Emergency, intensive care, and inpatient services of one private hospital  Period: 2015 | To determine the factors influencing MNC in hospitalised patients  Descriptive and correlational study | Convenience  Nurses: 71  Participation: 100%  Females: 77.5%  Baccalaureate nurses: 93.0%  Auxiliary nurses: 7%  Mean age: 28.4 yrs (SD 5.61 yrs)  1–2 yrs of work at the services: 47.9%  3–4 yrs of work at the institution: 35.2%  1–5 yrs of professional experience: 62% | Demographic characteristics: gender, age, nursing education, professional experience, number of patients assigned  Elements of MNC  Reasons for MNC  MISSCARE Survey (Part A and B), Spanish version |
| Hessels et al., 2015 [29]  New Jersey, United States  Acute care hospitals (n = 70)  Period: 2006 | To explore the relationship between specific factors of the nursing practice environment and MNC  Cross-sectional study | Randomised  Nurses: 7,679  Participation: 50%  Females: 97%  BSN: 44%  Specialty certification: 52% | Nurse participation in hospital affairs, nursing foundations for quality care, nurse manager ability, leadership, and support of nurses, staffing and resource adequacy, collegial nurse-physician relations  Elements of tasks left undone  Control variables: nurse staffing levels, hospital size, teaching status, high technology status, hospital geographic categories, nurse education  Practice Environment Scale of the Nursing Work Index  Tasks left undone |
| Higgs et al., 2016 [70]  Australia  Critical care/emergency specialty, medical, and surgical units in one tertiary referral hospital, Sydney  Period: N/A | To determine similarities and differences in elements of nursing care that are commonly rationed in the critical care, medical, and surgical specialties within an acute hospital environment  Cross-sectional study | Sampling method: N/A  RNs: 249  Participation: N/A  *Critical, medical, surgical unit*  Females: 81.6%, 88.5%, 93.2%  20–29 yrs: 43.7%, 42%, 44.4%  30–39 yrs: 21.8%, 31.8%, 23.6%  40–49 yrs: 17.2%, 13.6%, 20.8%  50–59 yrs: 14.9%, 8.0%, 5.6%  > 60 yrs: 2.2%, 4.5%, 5.6%  Less than a BSN: 11.9%, 9.1%, 9.7%  BSN: 48.8%, 75%, 80.6%  Graduate certificate/diploma: 25%, 9.1%, 8.3%  MSN or PhD: 14.3%, 6.8%, 1.4%  Mean yrs as RN: 11.5, 7.9, 8.1 | Demographic and background variables  Elements of MNC  MISSCARE scale (only Part A) |
| Kalisch & Lee, 2010 [52]  Midwestern United States  Medical/surgical, intermediate, intensive care, and rehabilitation units (n = 50) in four hospitals located in the Midwestern United States  Period: 2009 | To determine if the presence or absence of nursing teamwork results in MNC, which is any aspect of required patient care that is omitted or significantly delayed  Cross-sectional, descriptive study | Convenience  Nurses staff members: 2,216  Participation: 59.7%  Females: 89.2%  RNs: 76.5%  NAs: 22.2%  Licenses practical nurses: 1.3%  < 25 yrs: 14.7%; 26–34 yrs: 28.7%; 35–44 yrs: 26.7%; 45–54 yrs: 21.0%; > 55 yrs: 8.9%  High school grad: 14.7%  Associate degree: 38.5%  BSN: 42.6%  Graduate school: 4.2%  6 months to 2 yrs of experience: 23.7%; 2–5 yrs: 19.3%; 5–10 yrs: 17.7%; > 10 yrs: 34.1% | Nursing teamwork: trust, team orientation, backup, shared mental models, team leadership  Elements of MNC  Demographics: gender, age, education, experience, occupation, work schedules, perceptions of staff adequacy, overtime, absenteeism, staffing adequacy, number of hours of overtime  MISSCARE Survey (only Part A)  Nursing Teamwork Survey |
| Kalisch et al., 2011 [36]  Midwestern United States  Medical/surgical, rehabilitation, intermediate, and intensive care units (n = 110) in 10 acute care hospitals  Period: 2008–2009 | To examine the relationship between the levels and types of nurse staffing and MNC in acute care hospitals  Cross-sectional, descriptive study | Purposive  Nurses: 4,288  Participants: 60%  Females: 90%  RNs: 73.5%  > 35 yrs: 53.2%  BSN degree or higher: 46.7%  > 5 yrs experience: 51.0% | Staff characteristics: age, gender, education, experience in the profession/occupation, occupation, employment status, shift worked and its length, unit type, hours per patient days, registered nurse hours per patient day, skill mix, absenteeism, unit case mix index, work scheduled  MISSCARE Survey (only Part A) |
| Kalisch et al., 2011 [59]  Midwestern United States  Medical/surgical units, intensive care, intermediate care, and rehabilitation units of 10 hospitals  Period: 2008–2009 | 1. To identify the levels and types of MNC and reasons for MNC across hospitals  2. To examine the relationship between unit staff characteristics (gender, age, education, and experience in the role), work schedules (shift worked, length of shift, weekly worked hours, absenteeism, and unit type), staffing variables (perceived level of adequate staffing and number of patients cared for), and MNC  Cross-sectional study | Convenience  RNs: 3,143  NAs: 943  Participants: 59.8% (RNs 61.8%; NAs 53.4%)  Females: 90%  RNs: 77%  NAs: 23%  Baccalaureate degree or higher: 51%  Experience as nurse < 6 months: 5%  Experience as a nurse ≥ 10 yrs: 32% | Staff characteristics: education, job experience, gender, age  Work schedules: shift, hours worked  Staffing: absenteeism, perceived staffing adequacy, patient workloads  MISSCARE Survey (Part A and B) |
| Kalisch & Lee, 2012 [69]  Midwestern and Western United States  Medical/surgical, intermediate, intensive care, and rehabilitation units (n = 124) in 11 hospitals located in the Midwestern and Western United States  Period: 2008–2009 | To compare the amount, type, and reasons for MNC at Magnet and non-Magnet hospitals  Cross-sectional, descriptive study | Convenience  Nursing staff: 4,412  Participation: 57.3%  N/A | Magnet status of the hospitals  Elements of MNC  Reasons for MNC  Nursing education  Unit characteristics and type  Skill mix  Experience levels  MISSCARE Survey (Part A and B) |
| Kalisch et al., 2012 [86]  Two states, not specified  Units (n = 124) of 11 acute care hospitals  Period: 2008–2009 | To determine whether the omission of elements of nursing care leads to a greater number of patient falls, using actual fall rates and controlling for nurse staffing levels  Cross-sectional, descriptive study | Convenience  Nurses: 3,432  NAs: 980  Participation: 57.3%  Females: 91%  > 35 yrs: 54%  BSN or higher: 47%  > 5 yrs of experience: 51% | Elements of MNC items from the MISSCARE Survey: ambulation, patient assessments each shift, focused reassessment, response to call light, assistance with toileting  Hours per patient day  Fall rate (number of falls per 1,000 patient days) |
| Kalisch et al., 2013 [49]  Lebanon, Beirut, and Midwestern United States  United States: medical/surgical units, intermediate units, and ICUs (n = 14) in one teaching hospital  Lebanon: medical/surgical unit, intermediate unit, and ICUs (n = 18) in one teaching hospital  Period: N/A | To determine the extent of MNC and causes for it in Lebanon, comparing it with those in the United States  Descriptive, cross-sectional study | Convenience  U.S. nurses: 633  Lebanese nurses: 114  U.S. participants: 55.1%  Lebanese participants: 44.4%  *United States versus Lebanon*  Females: 90.8% versus 63.5%  > 35 yrs: 54.6% versus 8.7%  BSN: 56.2% versus 84.3%  > 5 yrs of experience: 48.7% versus 42.8%  > 10 yrs of experience: 14.4% versus 7.1% | Elements of MNC  Reasons for MNC: labour resources, material resources, communication  Staffing levels and job satisfaction: number of patients cared for in the last shift, satisfaction with current position, profession and teamwork  MISSCARE Survey (Part A and B) |
| Kim et al., 2018 [41]  South Korea  One tertiary university hospital  Period: 2017 | 1. To describe the levels of the nursing work environment, perception of the patient safety culture and MNC  2. To identify the inﬂuence of the work environment and patient safety culture on MNC  Cross-sectional study | Convenience  Nurses: 188 (the final sample was 186)  Participation: 98.9%  Females: 94.6%  Staff nurses: 70.7%  Charge nurse: 29.3%  Mean age: 28.36 yrs  Three-year diploma: 17.8%  BSN: 82.2%  ≥ MSN: 18.4%  Mean clinical career: 5.77 yrs | Nursing work environment: nurses’ participation in hospital affairs, nursing foundations for quality of care, nurse manager ability, leadership, and support of nurses, stafﬁng and resource adequacy, collegial nurse-physician relations  Patient safety culture: perception of patient safety culture, supervisor/manager, perception of communication on patient safety  Elements of MNC  Practice Environmental Scale of Nursing Work Index  Perception of Patient Safety Culture Scale  MISSCARE Survey (only Part A) |
| Labrague et al., 2019 [31]  The Philippines  Hospitals (n = 6) in the Central Philippines  Period: 2018–2019 | To examine the predictive role of nurse caring behaviours on MNC, adverse patient events, and the quality of nursing care  Cross-sectional study | Convenience  RNs: 600 (the final sample was 549)  Participation: 91.5%  Females: 78.7%  Staff nurse: 86.9%  Manager nurse: 13.1%  Mean age: 29.8 yrs  BSN: 91.1%  MSN/PhD: 8.9%  < 10 yrs in nursing: 81.2%; 10–19 yrs: 12.6%; >2 0 yrs: 6.2% | Caring behaviour of nurses  Elements of MNC  Adverse patient events: complaints from patients and their families, verbal abuse, falls, nosocomial infections, medication errors  Nurse-assessed quality of care  Caring Behaviour Inventory  MISSCARE survey (developed by Lake et al., 2017)  Adverse Patient Events Scale |
| Liu et al., 2018 [44]  China  Medical and surgical units (n = 111) in 23 hospitals  Period: 2014 | To explore the impact of hospital nursing organisational factors, including nurse work environment and workload, nursing care left undone, and nurse burnout, on patient safety in a Chinese context  Cross-sectional study | Convenience  Nurses: 1,671  Participation: 92.3%  Females: 98.8%  18–25 yrs: 40.1%; 25–30 yrs: 30.2%; 30–35 yrs: 16.0%; 35–40 yrs: 6.9%; 40–54 yrs: 6.8%  Secondary diploma: 53.2%  Advanced diploma: 37.3%  Baccalaureate degree and higher: 9.5%  < 5 yrs of experience: 52.0%; 5–10 yrs: 22.7%; 10–15 yrs: 12.0%; 15–20 yrs: 6.2%; 20–34 yrs: 7.1% | Demographic characteristics: gender, age, education level, years working in nursing  Nurse work environment: nurse participation in hospital aﬀairs, nursing foundation for quality of care, nurse manager ability, leadership, and support of nurses, staﬃng and resource adequacy, collegial nurse-physician relations  Nurses’ workload  Nursing care left undone  Nurse burnout  Patient safety: patient safety level, adverse events  Practice Work Environment Scale of the Nursing Work Index  Maslach Burnout Inventory-Human Services Survey |
| McNair et al., 2016 [57]  California, United States  Two hospitals (n = 15 units): University of California, Los Angeles, Santa Monica Medical Center (UCLA-SM) and University of California, San Francisco, Medical Center at Parnassus (UCSF-P)  Period:  UCLA-SM: 2012–2013  UCSF-P: 2013–2014 | 1. To examine the degree to which nurses reported that care was missed  2. To measure the time that RNs actually spent on various types of tasks  3. To examine the association between patterns of time use by nurses and reports of MNC at the level of the nursing unit  Longitudinal study  UCLA-SM: time 1, December 2012; time 2, September 2013  UCSF-P: time 1, April 2013; time 2, February 2014 | Randomised  Nurses: 669  Participation: 95.5% (639)  *UCLA-SM versus UCSF-P*  Female: 74.5% versus 73.2%  ≤ 30 yrs: 21.7% versus 34.6%  31–40 yrs: 45.0% versus 27.9%  41–50 yrs: 13.1% versus 23.6%  51–60 yrs: 12.5% versus 9.70%  ≥ 61 yrs: 1.99% versus 0.84%  > 6 yrs of experience: 48.7% versus 62.3% | Staff characteristics: nurse’s age, gender, years of nursing experience, use of NAs, numbers of patients cared for during the current shift  Elements of MNC  MISSCARE Survey (Part A and B) |
| Menard, 2014 [88]  New York, United States  Hospitals (n = 3) in rural western New York  Period: N/A | To investigate a potential relationship between workplace incivility and MNC  Descriptive, cross-sectional study | Convenience  RNs: 478 (the final sample was of 102)  Participation: 24.1%  Females: 90.2%  25–34 yrs: 33.3%; 35–44 yrs: 26.5%; 55–64 yrs: 13.7%  Associate degree in nursing: 58.8%  BSN: 34.3%  > 6 months to 2 yrs of experience: 16.7%; 2–5 yrs: 19.6%; 5–10 yrs: 25.5%; > 10 yrs: 37.3% | Elements of MNC  Reasons for MNC  Sources of incivility: inappropriate jokes, hostile climate, free-riding, abusive supervision, gossip/rumours, lack of respect, displaced frustration  MISSCARE Survey (Part A and B)  Nursing Incivility Scale |
| Nelson, 2017 [62]  Northwest Oregon, United States  Nursing home facilities (n = 16), both for-profit (n = 14) and non-profit (n = 2), Medicaid and/or Medicare certified  Period: 2016–2017 | 1. To determine the relationships between perceptions of workload, teamwork, and MNC as reported by nursing staff members in nursing home settings  2. To investigate whether teamwork is an operant mechanism through which workload is associated with MNC  Cross sectional, descriptive, correlational exploratory study | Convenience  Population: 139  Participants: 77.8%  Females: 83.5%  Charge nurses: 51.8%  Staff RNs: 15.1%  Staff licensed practical nurses: 25.2%  Certified nursing assistants: 7.9%  Mean age: 37.1 yrs (SD 10.9 yrs)  Associate degree: 46.0%  Baccalaureate degree: 39.6%  Graduate degree: 2.9%  Mean experience in current role: 6.3 yrs (SD 8.1 yrs) | Demographic and other information: age, gender, level of educational preparation, job title, role, experience in current role, unit and employer, sick leave use, overtime, intent to leave position, perception of staffing adequacy, normal shift worked  Unit and facility characteristics: number of residents and occupancy rate, profit status, size, staffing levels  Elements of MNC  Workload: nursing staff’s perception of workload as unit, unanticipated patient events, availability of support staff, all related to the past 3 months  Teamwork: trust, team orientation, backup, shared mental model, team leadership  MISSCARE Survey (only Part A)  Workload Subscale of the Individual Perceptions of Workload Scale  Nursing Teamwork Survey |
| Orique et al., 2016 [55]  California, United States  Acute care medical facility (n = 1)  Period: 2014 | Identify aspects of MNC and their relationship to unit-level nurse workload: types of MNC, reasons for MNC, types and reasons for MNC inﬂuenced by demographic characteristics, relationship between unit-level nurse workload, and incidence of MNC  Descriptive study | Convenience  RNs: 132  NAs: 25  Licensed vocational nurses: 12  Participation: N/A  Females: 85.2%  RNs: 78.1%  NAs: 14.8%  Licensed vocational nurses: 7.1%  25–34 yrs: 36.7% | Staff characteristics: demographic, work schedules, stafﬁng  Element of MNC  Reasons for MNC  MISSCARE Survey (Part A and B) |
| Palese et al., 2015 [67]  Italy  Acute medical units (n = 12)  Period: 2012 | 1. To identify the amount, type, and reasons for care being missed in the Italian medical care setting and to explore the factors that affect the occurrence of MNC  2. To describe the demographic and professional proﬁle of nursing staff working in medical units as well as their work satisfaction and intention to leave  Mixed-method approach: longitudinal survey and cross-sectional study (daily data collection for a period of three months) | Convenience  RNs: 252  NAs: 165  RNs participation: 81.3%  NAs participation: 66%  Females: 85%  < 25 yrs: 7.0%; 25–34 yrs: 28.3%; 35–44 yrs: 37.6%; > 45 yrs: 26.5%  Nursing diploma: 42.0%  University degree: 51.2%  Advanced education: 4.9%  2–5 yrs in current role: 21.7%  > 5 yrs in current role: 57.0% | Elements of MNC  Reasons for MNC  Demographic and professional data: age, gender, education, role occupied, length of experience in the professional role and in the medical ward, working time proﬁle, number of working hours per week, extra hours worked, shifts lost in the last 3 months, the number of patients cared for on the last shift, the number of admitted and discharged patients, intention to leave, satisfaction with the current role, the profession, and the team  MISSCARE Survey (Part A and B) |
| Papastavrou et al., 2014 [8]  Cyprus  Surgical (n = 211) and internal medicine (n = 156) units from all public general hospitals in Cyprus  Period: 2010–2011 | To explore the level and aspects of rationing of nursing care, and the potential relationship between nurses’ perception of their professional practice environment and rationing  Descriptive, correlational, cross-sectional multi-centre study | Convenience  Nurses: 715  Participation: 60.6%  Females: 71%  Mean age: 34.06 yrs  Nursing school diploma: 74.5%  University degree: 24%  MSN: 0.5%  Mean experience in nursing: 11.41 yrs (SD 9.27 yrs)  Mean experience in current unit: 5.32 yrs (SD 5.47 yrs) | Elements of MNC  Reasons for MNC  Nurse practice environment: handling disagreement and conflict, internal work motivation, control over practice, leadership and autonomy in clinical practice, staff relationships with physicians, teamwork, cultural sensitivity, communication about patients  Demographics: gender, age, educational level, employment status, number of years of experience in nursing  Basel Extent of Rationing of Nursing Care  Revised Professional Practice Environment scale |
| Papastavrou et al, 2016 [78]  Cyprus  All oncology and haematology units (n = 6) in Cyprus  Period: 2014 | To investigate nursing care rationing in oncology units: elements of care that are most often omitted, causes of MNC, any relationship between nursing care rationing, and intrinsic characteristics of nurses  Descriptive, co-relational, cross-sectional study | Convenience  RNs: 171  Participation: 91.8%  Females: 62.4%  25–34 yrs: 57.3%  > 2 yrs of experience: 57.1% | Staff characteristics: gender, age, education, hours of work, work experience, intention to leave, work satisfaction  Elements of MNC  Reasons for MNC  MISSCARE Survey (Part A and B) |
| Park et al., 2018 [66]  United States  1,583 units in acute care hospitals (n = 371)  Period: 2015 | 1. To examine the relationship between the quality of nurse practice environment and MNC  2. To identify which characteristics of the nurse practice environment are more likely to be associated with MNC  Descriptive, correlational study | Convenience  RNs: 31,650  Participants: 50%  N/A | Practice environment for nurses: nurse participation in hospital affairs, nursing foundations for quality of care, nurse manager ability, leadership and support of nurses, staffing and resource adequacy, collegial nurse–physician relations  Characteristics of the practice environment  Elements of MNC  Hospital and unit characteristics: hospital size, teaching status, location, Magnet status, patient case mix, unit type  Practice Environment Scale of the Nursing Work  MNC based upon the NDNQI RN |
| Phelan et al., 2018 [63]  Ireland  Nurses and Midwives Organisation  Period: 2015 | To examine the prevalence rates of MNC in the community nursing sector  Cross-sectional study | Purposive  Public Health Nurses: 1,500 (the final sample was 283)  Participation: 29%  Females: 98%  35–44 yrs: 34%  45–54 yrs: 34%  Primary degree or higher: 90%  6–15 yrs of work in community nursing: 59% | Demographic data  Components of community nursing: home nursing care, care management, family support, older people, disadvantaged groups, health promotion, education, provision of other community services, primary care teams, administration  Elements of MNC  Factors affecting MNC  Questionnaire based on the MISSCARE Survey (Part A and B) |
| Piscotty et al., 2014 [89]  Midwestern United States  Medical, surgical, intensive care, and intermediate care (n = 19) in one acute care hospital  Period: N/A | 1. To examine relationships between interventions supported by clinical decision support and reduced MNC  2. To examine relationships between nurses’ perceptions of health care information technology on their work and their reports of MNC  Descriptive study | Convenience  RNs: 165  Participation: 100%  Female: 87.9%  < 25 yrs: 13.9%; 25–34 yrs: 37.0%; 35–44 yrs: 23.0%; 45–54 yrs: 15.8%; 55–64 yrs: 9.7%; > 65 yrs: 0.6%  Associate degree: 26.7%  BSN: 69.1%  Graduate degree: 4.2%  < 6 months of experience: 4.8%; 6 month to 2 yrs: 24.2%; 2–5 yrs: 21.2%; 5–10 yrs: 13.9%; > 10 yrs: 35.8% | Nursing care reminders  Elements of MNC  Reasons for MNC  Demographics  Nursing care reminder usage survey  Impact of Healthcare Information Technology Scale  MISSCARE Survey (Part A and B) |
| Saqer et al., 2018 [87]  Jordan  Two governmental hospitals and two private hospitals  Period: 2016 | 1. To identify the types and reasons for MNC among Jordanian hospital nurses  2. To identify predictors of MNC based on background variables, confidence in delegation, and perceived reasons for MNC  3. To examine the relationship between nurses’ confidence in delegation and MNC  Cross-sectional study | Convenience  Nurses: 362  Participation: 78.1%  Females: 55.2%  Mean age: 29.5 yrs  BSN: 87.6%  MSN: 12.4%  ≤ 6 yrs of nursing experience: 50% | Elements of MNC  Reasons for MNC  Demographic factors (e.g. age, gender) and models of care delivery  Relationship between nurse confidence in delegation and MNC  Shift schemes (mixed shift, 8-hour or 12-hour shifts)  MISSCARE Survey (Part A and B)  The confidence and intent to delegate subscale |
| Schubert et al., 2013 [38]  Switzerland  Acute care hospitals (n = 35) from the German, French, and Italian language regions  Period: 2009–2010 | 1. To describe the levels of implicit rationing of nursing care in a quota sample of Swiss acute care hospitals  2. To explore the assumed associations between the quality of the nurse work environment dimensions, patient-to-nurse ratio, number of patients requiring support in all ADLs, number of patients requiring hourly or more frequent monitoring, patient safety climate, nurse experience, and education and implicit rationing of nursing care  Sub-study of the cross-sectional, multi-centre RN4CAST study, specifically the Swiss part | Convenience  RNs: 2,280  Participation: 71.6%  Females: 90%  Mean age: 35 yrs (SD 9.89 yrs)  BSN/MSN: 10%  Mean experience as a nurse: 8.00 yrs (SD 14.81 yrs)  Mean experience in this hospital: 5.00 yrs (SD 10.17 yrs) | Quality and elements of the nurse practice environment: nurse participation in hospital affairs, stafﬁng and resources adequacy, nurse foundations for quality of care, nurse manager ability, leadership support of nurses, collegial nurse–physician relations  Patient-to-nurse stafﬁng ratio  Nurse characteristics: experience, qualiﬁcation, age, sex, employment status  Hospital characteristics: typology, size  Revised version of Basel Extent of Rationing of Nursing Care instrument  Practice Environment Scale of the Nurse Work Environment Index-Revised  Safety Organizing Scale |
| Siqueira et al., 2017 [35]  Brazil  One large-scale teaching hospital  Period: N/A | To validate the MISSCARE BRASIL survey  Methodological and cross-sectional study | Simple randomisation  Nursing professionals: 330  Participation: N/A  Females: 77.3%  Aides: 39.7%  Technicians: 33%  Nurses: 20.9%  Nurses with administrative roles: 6.4%  Mean age: 39.9 yrs  Secondary education: 55.5%  Nursing technician school: 42.4%  > 10 yrs at the job: 52.1%  > 5 yrs of experience in the inpatient sector: 54.8% | Elements of MNC  Factors affecting MNC  MISSCARE Survey (Part A and B) |
| Smith et al., 2018 [71]  United States  Magnet (n = 3) and Pathway to Excellence (n = 2) to Excellence hospitals  Period: 2015 | 1. To describe the frequency of MNC in a multi-hospital U.S. sample  2. To determine the relationship between nurse work environments and MNC  3. To explore the association of the nurse work environment and collective efficacy with MNC  Quantitative, cross-sectional study | Convenience  RNs: 283  Participation: 8.1%  Females: 93%  20–25 yrs: 13%; 26–30 yrs: 22%; 31–40 yrs: 25%; 41–50 yrs: 20%; 51–75 yrs: 20%  < 1 year of experience: 12%; 1–2 yrs: 31%; 3–5 yrs: 18%; 6–10 yrs: 18%; 11–15 yrs: 10%; 16–20 yrs: 6%; >20 yrs: 5%  Associate degree: 18%  BSN: 74%  MSN: 6% | Demographics: age, years of experience on the unit, unit specialty  Nurse work environment: nurse manager ability, leadership, and support of nurses, nurse staffing and resource adequacy, nursing foundations for quality of care, nurse participation in hospital affairs, collegial nurse–physician relations  Elements of MNC  Collective efficacy  Practice Environment Scale of the Nurse Work Index  MISSCARE Survey (only Part A)  The Collective Efficacy Beliefs Scale |
| Srulovici et al., 2017 [43]  Israel  Different nursing units (internal medicine, surgery, intensive care, oncology, operating wards, psychiatry, paediatrics, obstetrics, and emergency units; n = 32) of eight public hospitals  Period: 2016 | To test the joint effects of personal and ward accountability on MNC, by using both focal (a nurse whose MNC is examined) and incoming (a nurse responsible for the same patients during the subsequent shift) nurses’ assessments of MNC  Cross-sectional study | Snowball  RNs: 172  Participation: 100%  Females: 75%  Mean age: 38.98 yrs (SD 9.58 yrs)  BSN: 69.78%  Mean professional experience: 13.93 yrs (SD 9.48 yrs) | Elements of MNC  Personal and organisational accountability  Nurse characteristics: age, gender, educational qualification, professional seniority, employment status  Workload: patient to nurse index, complexity of patients during the shift  MISSCARE survey (only part A) |
| VanFosson et al., 2018 [53]  United States  One 16-bed intensive care unit and one 24-bed progressive care unit of the U.S. Army Burn Center  Period: one week per month for six months | 1. To describe the monthly variation in the prevalence and patterns of unfinished nursing care  2. To determine the relationships between the nursing care system and unfinished nursing care  Repeated measures descriptive study | Convenience  Nurses: 118 (599 surveys)  Participation: 44.9%  Females: 66%  Licensed vocational nurses: 19%  RNs: 81%  Advanced individual training only: 1%  Some college: 16%  Associate degree: 28%  BSN: 49%  MSN: 6%  3 yrs of experience: 4%; 3–10 yrs: 35%; > 10 yrs: 61% | Demographics: unit type, shift worked, employment category, supply/demand ratio, patient turnover, overtime paid  Unfinished nursing care  Perceived Implicit Rationing of Nursing Care survey instrument |
| Vryonides et al., 2016 [77]  Cyprus  All oncology and haematology units (n = 6) in Cyprus  Period: 2014 | 1. To determine the different ethical climate types that are identified by nurses in cancer care units  2. To determine which type of ethical climate is prevalent  3. To investigate and describe the relationship (if any) between the identified types of ethical climates in cancer care units and the nurses’ perceived level of MNC  Descriptive correlation study | Convenience  RNs: 171  Participation: 91.8%  Females: 62.4%  < 34 yrs: 60.5%  BSN: 82.8%  MSN or PhD: 12.7%  Diploma: 4.5%  > 5 yrs of experience: 66.2% | Demographics: gender, age, workplace/care unit, level in nursing education, clinical experience  Ethical climate: caring, instrumental, independence, law and code, rules  Elements of MNC  Ethical Climate Questionnaire  MISSCARE survey-nurses version (only Part A) |
| White et al., 2019 [91]  California, Florida, Pennsylvania, and New Jersey, United States  Nursing homes (n = 540)  Period: 2015 | To examine how burnout and job dissatisfaction contribute to the likelihood of nursing home RNs leaving necessary care undone  Cross-sectional secondary study | Randomised  RNs: 231,000  Participation: 26%  Females: 92.7%  Mean age: 49.1 yrs  Hospital diploma: 14.8%  Associate degree: 46.7%  BSN: 36.0%  MSN or higher: 1.3%  Mean RNs experience: 16.6 yrs | Burnout  Job dissatisfaction: degree to which RNs were satisfied with their primary job and specific job aspects, health care, retirement, tuition benefits, salary/wages, work schedule, opportunities for advancement, independence at work, professional status  Elements of MNC  Nurse characteristics: age, years of RN experience, sex, race, native language, and highest nursing degree  Nursing home characteristics: ownership type, chain afﬁliation, bed size, payer mix, stafﬁng measures for RNs, licensed practical nurses, certiﬁed nursing assistants  Emotional Exhaustion subscale of the Maslach Burnout Inventory  MNC: nurses were asked to identify from a list of 14 care activities which, if any, were necessary but left undone due to lack of time or resources on their most recent shift/day worked |
| Winsett et al., 2016 [82]  United States  Medical, surgical, or combined medical/surgical units (n = 18) in four non-academic medical centres  Period: 2014 | 1. To examine the nurse work environment by evaluating the self-reported MNC and reasons for MNC from nurses on medical surgical units  2. To describe the frequency and reasons for MNC  3. To describe the relationships among the unit types for frequency of MNC  Descriptive correlational study | Convenience  Nurses: 586  Participation: 29%  Age: 36 ± 12.6 yrs  BSN: 40.5%  Diploma: 7.1%  MSN: 4.2%  6 months to 2 yrs of experience: 28.6%; > 10 yrs: 33.4%  6 months to 2 yrs in current unit: 34.0%; > 10 yrs: 22.0% | Demographic characteristics: age, educational degree, primary shift worked, stafﬁng adequacy, usual number of hours worked per week, overtime hours and missed shifts in the previous three months, number of patients assigned during last shift worked with number of admissions and discharges  Unit characteristics: total unit full time equivalents, RN hours per patient day, case mix index, skill mix  Element of MNC  Reasons for MNC  MISSCARE Survey (Part A and B) |
| Zander et al., 2014 [84]  Germany  Hospitals (n = 49)  Period: 2009–2011 | To describe the prevalence and patterns of nursing care left undone as well as its association with the nurse work environment and staffing in German acute care hospitals (as part of the RN4CAST Study)  Cross-sectional, descriptive study | Convenience  Professional nurses: 1,511  Participation: 44%  Females: 89.3%  More than 10 yrs of professional experience: 68.6%  Average yrs of experience: 10.3 yrs | Quality of nurse work environment  Nurse staffing level  Nurse factors: age, gender, employment level; level of emotional exhaustion  RN4CAST nurse questionnaire |
| Zhu et al., 2019 [43]  China  Hospital medical and surgical units (n = 181) from nine provinces, municipalities, and autonomous regions in mainland China  Period: N/A | To explore the interrelationships among the diﬀerent aspects within nursing work systems using structural equation modelling  Cross-sectional study | Convenience  Nurses: 7,802  Patients: 5,430  Participation: N/A  Female nurses: 99.50%  Mean nurse age: 29.42 yrs  Mean working yrs as a nurse: 8.73  Secondary diploma (nurses): 18.41%  Advanced diploma (nurses): 61.28%  BSN higher (nurses): 20.31%  Mean patient age: 54.24  Mean patients length of stay: 14.83 days | Patient outcome indicators: nurse-reported quality assessments, patient adverse events, patient-reported dissatisfaction with hospital care  Rationing of nursing care: comfort/talk with patients, teach/counsel patients and family, adequate patient surveillance, prepare patients and families for discharge, coordinating patient care, develop or update nursing care pans, skin care, pain management, adequately document nursing care, oral hygiene, treatments and procedures, administer medications on time  Characteristics of hospital organisation and unit type  Nurses education and working time  Patient demographics: length of stay, self-rated health status, educational level  China Nurse Survey  Basel Extent of Rationing of Nursing Care |
| Zúñiga et al., 2015 [50]  Switzerland  Nursing home facilities from all three language regions in Switzerland (n = 156), nursing home facilities (n = 402), and teams not bound to a speciﬁc unit (n = 74)  Period: 2012–2013 | 1. To describe levels and patterns of self-reported implicit rationing of care  2. To explore the relationship between stafﬁng level, turnover, and work environment factors and implicit rationing of nursing care  Sub-study of the Swiss Nursing Home Human Resources Project | Random  Care workers: 4,307  Participation: 78%  Females: 92.3%  RNs: 25.3%  Licensed practical nurses: 21.5%  Certiﬁed assistant nurses: 19.8%  Nurse’s aides: 30.1%  Other 3.3% | Elements of implicit rationing of nursing care  Leadership: nurse manager ability, leadership, support of care workers  Stafﬁng and resources adequacy  Teamwork and resident safety climate  Work stressors: workload, conflict and lack of recognition, lack of preparation  Demographics: gender, age, usual shift, educational background  Unit characteristics  Resident characteristics: age, length of stay, resident care load  Basel Extent of Rationing of Nursing Care instrument  Two subscales of the Practice Environment Scale–Nursing Work Index  The Safety Attitudes Questionnaire  Health Professions Stress Inventory |
| **Cohort studies** |  |  |  |
| Griffiths et al., 2018 [32]  England  Adult medical and surgical wards (n = 32) of a large acute general hospital  Period: 2012–2015 | 1. To determine whether adverse outcomes occur after patients are exposed to low nurse staffing levels on hospital wards, and whether missed observations mediate this relationship and could thus provide a useful indicator of inadequate staffing levels  2. To examine whether, and how, variation in nurse staffing levels on general hospital wards is associated with omissions or delays in delivering necessary nursing care  3. To model the possible costs and consequences of changes in staffing levels  4. To provide a basis for identifying the nurse staffing levels and skill mix required to ensure adequate patient surveillance, and to assess whether rates of missed vital signs observations can be used to identify when or where care is falling below accepted standards and putting patients at risk  Retrospective, longitudinal observational study (time 0, admission date; ending time, indicator for death) | Convenience  Patients: 138,133 (294,5265 complete observations)  Participation: N/A  Females: 53%  Mean age: 67 yrs (SD 20.61 yrs)  < 65 yrs: 47%; 65–74 yrs: 18%; 75–85 yrs: 21%; ≥ 85 yrs: 14%  Patients died: 4.1%  Average skill mix: 60% RN | Adverse event outcome: death, cardiac arrest or unplanned ICU admission  Missed observations  Vital signs observations  Nutritional risk assessments  Nursing staff data  Nutritional risk with Malnutrition Universal Screening Tool  National Early Warning Score |
| Hogh et al., 2018 [30]  Denmark  Municipalities: eldercare sector (n = 10)  Period: time 1, 2006; time 2, 2008 | 1. To analyse the long-term impact of bullying among health care providers (time 1) on MNC and quality of care 2 yrs later (time 2)  2. To test the potential mediating effect of affective organisational commitment  Prospective cohort study (time 1 and time 2) | Convenience  Health care providers engaged in provision of care: 4,000 at time 1 clustered; N/A at time 2  Females: 97.6% | Bullying: if respondents had been exposed to bullying within the past 12 months and how often  Mediator  Covariates: place of work, tenure at current job, professional level  MNC with a two-item scale (‘How often does it happen that the allocated time is not sufficient to meet the needs of the client?’ and ‘How often do you have to finish a visit with a client with the feeling that you have not done what was necessary?’) |
| Knopp-Sihota et al., 2015 [31]  Canada  Nursing homes (n = 36)  Period: 2010 | 1. To describe the nature and frequency of rushed or missed care by health care aides  2. To assess the association of rushed or missed care with care aide characteristics or work characteristics such as organisational context at the nursing home microsystems level  Longitudinal study | Random stratified  Health care aides: 583  Participation: N/A  Females: 94.2%  < 30 yrs: 13.0%; 30–39 yrs: 22.3%; 40–49 yrs: 32.1%; 50–59 yrs: 23.7%; > 60 yrs: 8.9%  Mean yrs worked as a care aide: 11 (SD 8.7) | Demographic variables: age, sex, years worked as care aide, shift worked most often  Job satisfaction and vocational satisfaction  Mental and physical health status  Burnout  Organisational context: province, location, size and owner/operator model  Outcome variables: times felt rushed and missed resident care  Missed Resident Care  SF-8^TM^ Health Survey  Maslach Burnout Inventory  Alberta Context Tool  Missed Resident Care |
| **Quasi-experimental study** |  |  |  |
| Kalisch et al., 2013 [34]  United States  Medical/surgical units (n = 3) in three acute care hospitals  Period: N/A | To test the impact of a train-the-trainer intervention on the level of satisfaction with nursing teamwork and the amount of MNC  Quasi-experimental study (time 1, time 2, and time 3 are the pre-test, post-test, and 2 months after completion of the intervention, respectively) | Convenience  Nursing staff: 242  Participation: 83.1% for the pre-test surveys, 84.4% for the post test, 73.3% for the follow-up  Females: 89.5%  RNs: 65%  NAs: 30%  Unit secretaries: 4%  Nursing/assistant manager: 1.7%  Licensed practical nurses: 1.2%  Age > 45 yrs: 32.5%  Licensed practical nurse diploma: 3.6%  RNs diploma: 6.6%  Associate degree: 29.9%  BSN: 49.1%  Bachelor outside of nursing: 6.0%  MSN or higher in nursing: 4.8%  < 6 months of experience: 7.5%; 6 months to 2 yrs: 14.1%; 2–5 yrs: 19.1%; 5–10 yrs: 26.1% | Nursing teamwork: trust, team orientation, backup, shared mental models, team leadership  Satisfaction with teamwork  Knowledge of teamwork  Elements of MNC  Nursing Teamwork Survey  MISSCARE Survey (Part A and B)  Knowledge of Teamwork modification of the knowledge test contained in the Agency of Healthcare Research and Quality Team STEPPS instructor guide |

**Abbreviations:** ADL: activity of daily living; BSN: bachelor of science in nursing; CI: confidence interval; GED: general equivalency diploma; ICU: intensive care unit; IRR: incident rate ratio; MNC: missed nursing care; MSN: master of science in nursing; NA: nursing assistant; NDNQI: National database of Nursing Quality Indicators; N/A: not available; PhD: doctor of philosophy; RN: registered nurse; RN4CAST: Registered Nursing Forecasting; SD: standard deviation; yrs: years.

**Supplementary file 2**

|  | Al-Kandari et al., 2009 [54] | Ausserhofer et al, 2014 [46] | Ball et al., 2014 [39] | Ball et al., 2016 [85] | Ball et al., 2018 [47] | Bekker et al., 2015 [58] | Blackman et al., 2014 [37] | Blackman et al., 2017 [48] | Blackman et al., 2018 [60] | Blackman et al., 2019 [11] | Bragadòttir et al., 2016 [64] | Castner et al., 2014 [56] | Chapman et al., 2016 [61] | Cho et al., 2015 [81] | Cho et al., 2016 [40] | Coleman, 2018 [68] | Dhaini et al., 2017 [51] | Drach-Zahavy & Srulovici, 2019 [42] | Duffy et al., 2018 [65] | Friese et al., 2013 [83] | Hernández-Cruz et al., 2017 [90] | Hessels et al., 2015 [29] | Higgs et al., 2016 [70] | Kalisch et al., 2010 [52] | Kalisch et al., 2011 [36] | Kalisch et al., 2011 [59] | Kalisch & Lee, 2012 [69] |
| --- | --- | --- | --- | --- | --- | --- | --- | --- | --- | --- | --- | --- | --- | --- | --- | --- | --- | --- | --- | --- | --- | --- | --- | --- | --- | --- | --- |
| **Item 1**. Were the criteria for inclusion in the sample clearly defined? | U | Y | Y | U | Y | Y | Y | Y | Y | N | Y | Y | Y | Y | N | Y | Y | N | Y | U | U | N | N | N | Y | Y | Y |
| **Item 2**. Were the study subjects and the setting described in detail? | N | Y | Y | U | Y | Y | U | Y | N | N | Y | Y | Y | Y | Y | Y | Y | N | Y | U | N | U | N | Y | Y | Y | Y |
| **Item 3**. Was the exposure measured in a valid and reliable way? | N | Y | Y | U | N | N | Y | Y | Y | Y | Y | Y | Y | U | U | Y | Y | U | Y | N | Y | Y | N | Y | N | N | Y |
| **Item 4**. Were objective, standard criteria used for measurement of the condition? | N | Y | Y | Y | Y | Y | Y | U | U | U | U | Y | Y | N | Y | Y | Y | Y | U | N | N | Y | U | Y | Y | Y | Y |
| **Item 5**. Were confounding factors identified? | N | Y | N | N | U | N | N | N | U | N | N | N | N | N | N | N | Y | N | N | N | N | Y | N | U | N | N | N |
| **Item 6**. Were strategies to deal with confounding factors stated? | NA | Y | N | N | NA | N | NA | NA | U | NA | N | NA | N | NA | N | N | Y | NA | NA | N | NA | Y | N | Y | N | NA | NA |
| **Item 7**. Were the outcomes measured in a valid and reliable way? | N | Y | Y | Y | N | U | Y | Y | Y | U | Y | Y | Y | Y | U | Y | Y | U | Y | Y | Y | Y | Y | Y | Y | Y | U |
| **Item 8**. Was appropriate statistical analysis used? | Y | Y | Y | Y | Y | Y | Y | Y | Y | Y | Y | Y | Y | Y | Y | Y | Y | Y | Y | Y | Y | Y | Y | Y | Y | Y | Y |

Quality assessment of included studies: critical appraisal tool for analytical cross-sectional studies [26]

**Abbreviations:** Y: yes; N: no; U: unclear; NA: not applicable.

‡Sampling, data collection, and data management were reported elsewhere.

|  | Kalisch et al., 2012 [86] | Kalisch et al., 2013 [49] | Kim et al., 2018 [41] | Labrague et al., 2019 [31] | Liu et al., 2018 [44] | McNair et al., 2016 [57] | Menard, 2014 [88] | Nelson, 2017 [62] | Orique et al., 2016 [55] | Palese et al., 2015 [67] | Papastavrou et al., 2014 [8] | Papastavrou et al, 2016 [78] | Park et al., 2018 [66] | Phelan et al., 2018 [63] | Piscotty et al., 2014 [89] | Saqer et al., 2018 [87] | Schubert et al., 2013 [38] | Siqueira et al., 2017 [35] | Smith et al., 2018 [71] | Srulovici et al., 2017 [43] | VanFosson et al., 2018 [53] | Vryonides et al., 2016 [77] | White et al., 2019 [91] | Winsett et al., 2016 [82] | Zander et al., 2014 [84] | Zhu et al., 2019 [43] | Zúñiga et al., 2015 [50] |
| --- | --- | --- | --- | --- | --- | --- | --- | --- | --- | --- | --- | --- | --- | --- | --- | --- | --- | --- | --- | --- | --- | --- | --- | --- | --- | --- | --- |
| **Item 1**. Were the criteria for inclusion in the sample clearly defined? | Y | U | Y | Y | N | Y | Y | Y | Y | Y | Y | Y | Y | U | Y | Y | Y | Y | Y | Y | Y | N | U‡ | Y | U | N | Y |
| **Item 2**. Were the study subjects and the setting described in detail? | Y | Y | N | Y | U | Y | Y | Y | Y | Y | Y | Y | Y | N | Y | Y | Y | U | Y | Y | Y | N | U‡ | Y | Y | Y | Y |
| **Item 3**. Was the exposure measured in a valid and reliable way? | Y | N | Y | NA | Y | N | Y | Y | U | Y | U | N | Y | NA | Y | U | Y | NA | Y | Y | N | N | N | N | U | N | Y |
| **Item 4**. Were objective, standard criteria used for measurement of the condition? | Y | Y | Y | Y | Y | Y | Y | Y | U | Y | Y | U | Y | NA | Y | N | Y | Y | Y | Y | Y | U | Y | U | U | Y | Y |
| **Item 5**. Were confounding factors identified? | N | N | N | N | N | N | U | N | N | N | U | N | U | N | N | N | Y | NA | N | N | N | N | N | N | Y | Y | Y |
| **Item 6**. Were strategies to deal with confounding factors stated? | N | N | NA | NA | N | NA | U | N | N | N | Y | N | NA | N | N | NA | Y | NA | N | NA | NA | N | NA | N | Y | Y | Y |
| **Item 7**. Were the outcomes measured in a valid and reliable way? | N | Y | Y | U | Y | N | Y | Y | Y | Y | U | Y | N | U | Y | Y | Y | NA | Y | Y | Y | Y | U | Y | N | Y | Y |
| **Item 8**. Was appropriate statistical analysis used? | Y | Y | Y | Y | Y | Y | Y | Y | U | Y | Y | Y | Y | Y | Y | Y | Y | Y | Y | Y | U | Y | Y | Y | Y | Y | Y |

**Abbreviations:** Y: yes; N: no; U: unclear; NA: not applicable.

‡Sampling, data collection and data management were reported elsewhere.

**Supplementary file 3**

Quality assessment of the included studies: critical appraisal tool for cohort studies [27]

|  | Griffiths et al., 2018 [32] | Hogh et al., 2018 [30] | Knopp-Sihota et al., 2015 [31] |
| --- | --- | --- | --- |
| **Item 1**. Were the two groups similar and recruited from the same population? | NA | Y | Y |
| **Item 2**. Were the exposures measured similarly to assign people  to both exposed and unexposed groups? | NA | Y | U |
| **Item 3**. Was the exposure measured in a valid and reliable way? | Y | N | Y |
| **Item 4**. Were confounding factors identified? | Y | Y | N |
| **Item 5**. Were strategies to deal with confounding factors stated? | Y | Y | NA |
| **Item 6**. Were the groups/participants free of the outcome at the start of the study (or at the moment of exposure)? | NA | NA | NA |
| **Item 7**. Were the outcomes measured in a valid and reliable way? | Y | N | U |
| **Item 8**. Was the follow up time reported and sufficient to be long enough for outcomes to occur? | NA | Y | NA |
| **Item 9.** Was follow up complete, and if not, were the reasons to loss to follow up described and explored? | NA | Y | NA |
| **Item 10.** Were strategies to address incomplete follow up utilised? | NA | N | NA |
| **Item 11.** Was appropriate statistical analysis used? | Y | Y | Y |

**Abbreviations***:* Y: yes; N: no; U: unclear; NA: not applicable.

**Supplementary file 4**

Quality assessment of the included studies: critical appraisal tool for quasi-experimental studies (non-randomised experimental studies) [28]

|  | Kalisch et al., 2013 [34] |
| --- | --- |
| **Item 1**. Is it clear in the study what is the ‘cause’ and what is the ‘effect’ (i.e. there is no confusion about which variable comes first)? | Y |
| **Item 2**. Were the participants included in any comparisons similar? | Y |
| **Item 3**. Were the participants included in any comparisons receiving similar treatment/care, other than the exposure or intervention of interest? | NA |
| **Item 4**. Was there a control group? | N† |
| **Item 5**. Were there multiple measurements of the outcome both pre- and post-the intervention/exposure? | Y |
| **Item 6**. Was follow up complete and if not, were differences between groups in terms of their follow up adequately described and analysed? | Y |
| **Item 7**. Were the outcomes of participants included in any comparisons measured in the same way? | Y |
| **Item 8**. Were outcomes measured in a reliable way? | Y |
| **Item 9.** Was appropriate statistical analysis used? | Y |

**Abbreviations:** Y: yes; N: no; U: unclear; NA: not applicable.

†An independent control group was not involved in the study.
